# Egg White-Based Biomaterials: Morphological Architectures and Emerging Biomedical Applications

**DOI:** 10.3390/polym18202550

**Published:** 2026-10-16

**Authors:** Hatice Asri, Fatma Zehra Köse, Mohammadreza Ghaffarlou, Ahmet Erdem

**Affiliations:** 1 Department of Biomedical Engineering, Umuttepe Campus, Kocaeli University, Kocaeli 41001, Türkiye; 2 Department of Biomedical Engineering, Istanbul Yeni Yuzyil University, Zeytinburnu, Istanbul 34010, Türkiye; 3 Department of Polymer Science and Technology, Umuttepe Campus, Kocaeli University, Kocaeli 41001, Türkiye

**Keywords:** EW, ovalbumin, natural biomaterials, hydrogels, tissue engineering, drug delivery, 3D bioprinting, structure–property relationship

## Abstract

Egg white (EW) has emerged as a versatile natural biomaterial due to its wide availability, low cost, biodegradability, intrinsic bioactivity, and protein-derived gel- and film-forming capabilities. However, the existing literature has largely addressed EW-based biomaterials in terms of individual fabrication methods or specific applications, while comprehensive evaluations linking processing strategies to the resulting morphology, physicochemical properties, and biomedical functions remain limited. In this review, EW-based biomaterials are critically evaluated through an integrated processing, structure, property, and function framework encompassing molecular composition, fabrication strategies, morphological organization, physicochemical behavior, and biomedical performance. The molecular interactions and processing-dependent structural changes in major EW proteins, including ovalbumin, ovotransferrin, ovomucoid, ovomucin, and lysozyme, are discussed, and physical gelation, chemical and enzymatic crosslinking, chemical functionalization, and polymer/nanomaterial hybridization are compared as major fabrication approaches. These strategies enable the development of diverse morphologies, including hydrogels, nano/microparticles, films and membranes, micro/nanofibers, porous sponges, cryogels, and 3D-printed constructs, which provide distinct functional advantages in tissue engineering, 3D cell culture, wound healing, drug and bioactive molecule delivery, bioprinting, and biosensing. Particular emphasis is placed on the roles of crosslink density, porosity, swelling behavior, mechanical properties, degradation, and molecular transport in governing cellular responses and overall biomedical performance. Despite these advantages, batch-to-batch variability, the limited mechanical robustness of native protein networks, sterilization and storage constraints, and potential allergenicity and immunogenicity remain major barriers to clinical translation. Future research should therefore focus on standardized protein formulations, application-specific hybrid networks, advanced biofabrication approaches, and comprehensive long-term biosafety studies to enhance the translational potential of EW-based biomaterials.

## 1. Introduction

Naturally derived biomaterials have attracted increasing attention as alternatives to synthetic materials for biomedical applications, including tissue engineering, regenerative medicine, wound healing, and drug delivery, owing to their biocompatibility, biodegradability, intrinsic biological activity, and renewability [1]. Among these natural resources, eggs and their individual components have gained considerable interest as accessible, low-cost, and compositionally rich sources of bioactive molecules for biomaterial development [2]. Distinct egg components have been explored for the fabrication of diverse biomedical platforms, including hydroxyapatite-based materials, protein-derived scaffolds, wound dressings, hydrogels, bioprinting systems, and biosensors, exploiting their distinctive chemical, structural, and biological characteristics [2,3,4]. Thus, rather than being considered simply as a source of individual biomaterials, eggs can be viewed as a multifunctional natural biomaterial platform comprising components with distinct structural and biochemical properties.

An egg is composed primarily of the eggshell, egg membrane, EW, and egg yolk, each of which has distinct structural and biochemical features relevant to biomedical applications. In particular, the eggshell, which is rich in calcium carbonate, has been investigated as a natural calcium source for the fabrication of hydroxyapatite and other calcium phosphate-based materials for bone tissue engineering [5,6]. The egg membrane, owing to its fibrous protein-rich structure, has attracted interest in wound healing and tissue regeneration [7,8]. Similarly, egg yolk, which contains lipids, proteins, minerals, and immunoglobulin Y (IgY), has been investigated for applications in wound healing, tissue engineering, and diagnostic and therapeutic systems [9]. Among these components, however, EW is particularly attractive for direct biomaterial development because of its abundant protein content, intrinsic biological functionality, and versatile protein gelation and assembly behavior, which can be exploited through a range of physicochemical and biochemical processing strategies.

EW accounts for approximately 58% of the egg and is composed primarily of proteins such as ovalbumin, ovotransferrin, ovomucoid, ovomucin, and lysozyme [1,10]. These proteins are important not only nutritionally but also because of their functional properties, including surface activity, water-holding capacity, film-forming ability, emulsification, and gelation. Among them, ovalbumin is the predominant EW protein and can form protein–protein interactions and network structures in response to stimuli such as heat, pH, ionic strength, and chemical modification, making EW particularly advantageous for biomaterial production. Ovalbumin is also widely used as a natural, biocompatible, and biodegradable protein polymer in the development of hydrogels, particulate systems, and delivery systems for bioactive substances [11].

The potential of EW as a biomaterial lies in the ability of its protein components to participate in a range of molecular interactions and crosslinking mechanisms. Hydrogen bonding, hydrophobic and electrostatic interactions, and disulfide bonding are important in the assembly of proteins into three-dimensional network structures, while physical and chemical crosslinking approaches allow the structural integrity and properties of these networks to be controlled. Protein concentration, pH, temperature, ionic environment, and crosslinking conditions influence protein conformation, aggregation, and network formation, thereby determining the final structure of the resulting biomaterial. Therefore, the biomaterial performance of EW depends not only on its protein composition but also on the molecular interactions exhibited by these components under processing conditions [4,9,12].

This structural versatility enables EW and its components to be transformed into biomaterial platforms with diverse morphological and physicochemical properties. Various EW-based structures, including hydrogels, nanoparticles, microparticles, aerogels, thin films, and three-dimensional porous scaffolds, have been reported in the literature, and their potential biomedical applications, such as wound healing, delivery of drugs and bioactive compounds, tissue engineering, 3D bioprinting, and biosensing, have been explored [12,13]. The morphology, porosity, mechanical properties, swelling and degradation behavior, and cell–material interactions of these structures can vary considerably depending on the protein components used and the fabrication strategy employed. Consequently, biomaterial forms derived from the same natural protein source can offer distinct structural and functional advantages according to their intended application [9].

Egg-derived biomaterials have previously been addressed in several reviews [4,9,14], which examined the eggshell, shell membrane, egg white, and egg yolk collectively, focusing on tissue engineering and general biomedical applications; in these reviews, EW was treated as one of several egg components rather than examined in detail. Reviews focusing exclusively on EW, such as [2,13], described the composition, biological properties, resulting morphologies, and biomedical uses of EW, whereas [13,15] also addressed non-biomedical applications such as packaging films, bioplastics, and bioceramics. To our knowledge, these two studies are the most recent reviews (2020–2021) focusing specifically on EW-based biomedical materials. Since 2021, the field has expanded considerably, with the development of photocrosslinkable EW derivatives [16], enzymatically and nanomaterial-reinforced networks [3,17,18], hierarchically structured scaffolds [19], and cell- and vesicle-loaded EW systems [20,21]. Nevertheless, a comprehensive evaluation of the relationships among the fabrication methods, morphological architectures, structural properties, crosslinking mechanisms, and biomedical functions of EW-based materials remains limited [2,4,9,13,14]. In particular, systematic comparisons of different EW-based morphologies in terms of fabrication, material properties, and biological performance are lacking, and translational aspects such as reproducibility, sterilization, and immunogenicity have received limited attention. To address these gaps, studies published primarily between 2021 and 2026 are evaluated herein.

**Figure 1:**
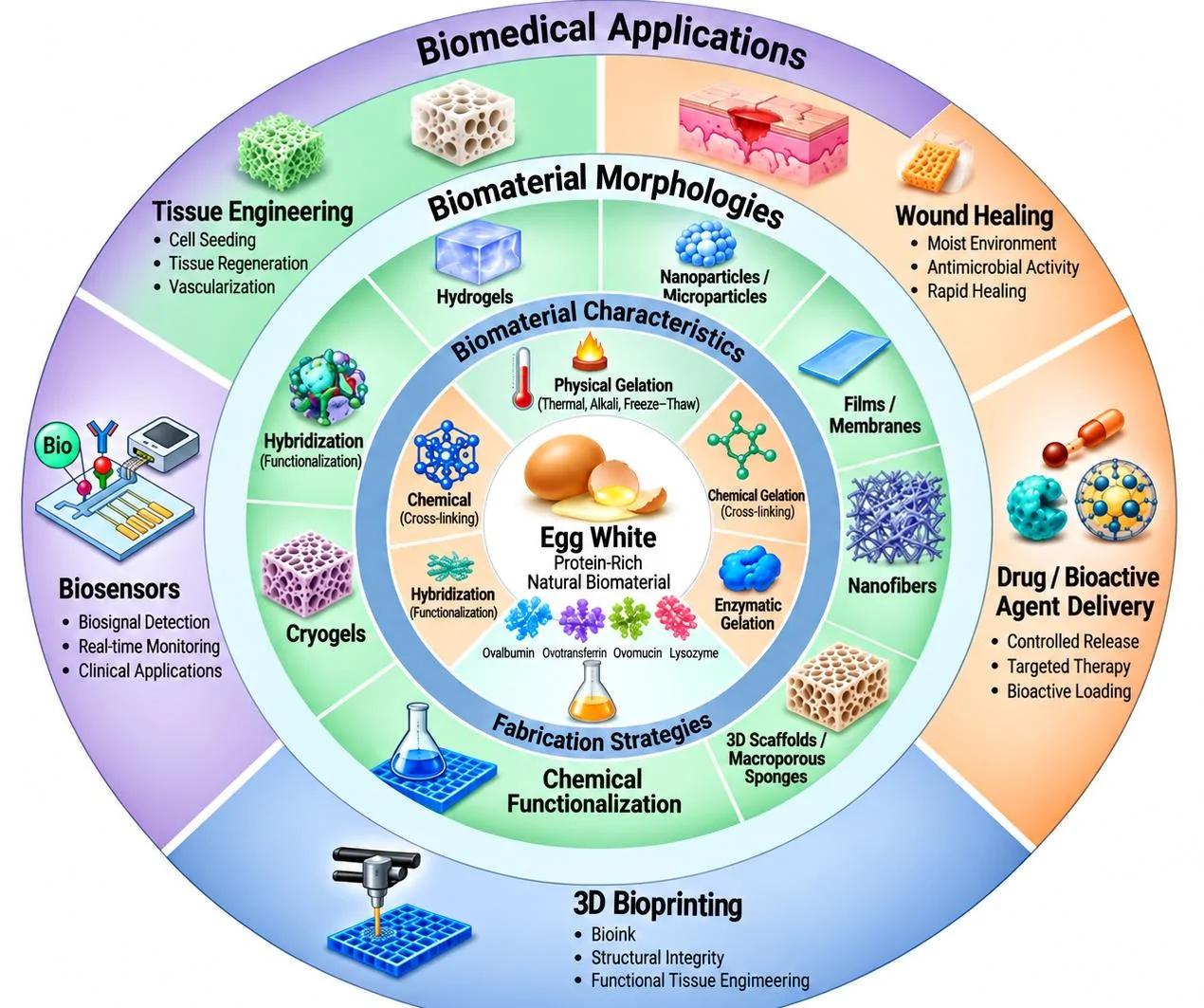
Egg-white-based biomedical applications.

This review examines the potential of EW as a source of biomaterials for biomedical applications, with particular emphasis on morphology and structure–function relationships. It compares physical and chemical methods used to convert EW proteins into different forms, mainly hydrogels, nanoparticles, and thin films, and evaluates how functional groups, molecular interactions, gelation, and crosslinking influence their structural and physicochemical properties. The review also assesses the performance of these biomaterials in wound healing, tissue engineering, drug and bioactive delivery, 3D bioprinting, and biosensing. Finally, it highlights the limitations of current studies and outlines future research directions needed to develop more controlled, functional, and clinically applicable EW-based biomaterials (Figure 1).

This review is based on a literature search conducted on Google Scholar and Web of Science up to 1 July 2026. The terms egg white, ovalbumin, and egg white protein were combined with keywords related to biomaterials, hydrogels, scaffolds, crosslinking, bioprinting, wound healing, drug delivery, and biosensors. The study includes research in which EW or EW-derived proteins served as the primary material component and where data regarding production, structure, or biological properties were presented. Within this scope, foundational earlier studies addressing the basic structural or gelation characteristics of EW-based biomaterials were also evaluated. Food science studies were included in the assessment to provide a comprehensive overview of the fundamental gelation behavior and network formation principles of EW proteins. Publications in which EW proteins were investigated solely as an analytical target were excluded.

## 2. Structure of EW and Factors Affecting Its Interaction

EW is a complex biological system composed of approximately 88% water and 10–11% protein, together with smaller amounts of carbohydrates, minerals, and lipids [11]. Its protein fraction consists mainly of ovalbumin (54%), ovotransferrin (12%), ovomucoid (11%), globulins (8%), ovomucin (3.5%), and lysozyme (3.4–3.5%). Although ovalbumin is the predominant protein, ovotransferrin, ovomucin, and lysozyme also contribute substantially to the functional properties of EW, including gel formation, viscoelastic behavior, and biological activity [13,22]. In particular, the metal ion-binding capacity of ovotransferrin and the natural antimicrobial activity of lysozyme indicate that EW is not only a structural protein source but also a natural biomaterial platform with important biological functions.

The behavior of EW proteins during biomaterial formation is closely associated with their molecular structures and the chemical functionalities of their constituent amino acid residues. Protein–protein interactions and network formation are driven by terminal amino and carboxyl groups, as well as polar, ionizable, hydrophobic, and reactive side chains. In particular, residues such as serine, tyrosine, and cysteine participate through hydrogen bonding, electrostatic and hydrophobic forces, and disulfide bond dynamics. Sulfhydryl/disulfide groups derived from cysteine play a crucial role in intermolecular bond formation during protein denaturation and aggregation. These molecular interactions enable proteins to assemble into three-dimensional networks, allowing EW to form gel-like or more organized biomaterial structures under various physicochemical conditions. Therefore, the final properties of EW-based biomaterials depend not only on protein composition but also on the conformational changes and intermolecular interactions that occur during processing [13,22].

Parameters such as pH, temperature, protein concentration, and ionic environment play a decisive role in the structural organization of EW proteins. Changes in pH alter the net surface charge of proteins and, consequently, the electrostatic repulsive or attractive forces between molecules. As pH approaches the isoelectric points of isolated proteins, reduced electrostatic repulsion can enhance protein–protein interactions and promote aggregation. However, the resulting gel morphology and mechanical properties depend not only on pH but also on the combined effects of protein concentration, heat treatment, and ionic conditions. Indeed, recent studies on the gelling properties of EW proteins have shown that pH is a key variable influencing aggregation, gel network formation, and final gel properties through effects on protein charge and conformation [22,23]. Experimental studies have also demonstrated that gel morphology and water retention behavior can vary under different pH conditions [22].

Temperature is a critical parameter in regulating protein denaturation and subsequent aggregation and gelation processes. During heat treatment, native protein conformations partially unfold, exposing buried hydrophobic regions and reactive functional groups, thereby increasing protein–protein interactions. In EW, the initial thermal transition occurring at approximately 61.5–62.5 °C is associated with the denaturation of less thermally stable proteins, primarily ovotransferrin, whereas at higher temperatures, ovalbumin and other proteins continue to denature and aggregate, contributing to the formation of the main gel network [22]. Therefore, temperature should be regarded not only as a factor that initiates gelation but also as a fundamental process variable that influences protein conformation, aggregation kinetics, and the microstructure of the resulting network.

The ionic environment and protein concentration also play a significant role in the formation of EW-based structures. Ions can alter electrostatic interactions between proteins and thereby influence their aggregation behavior, while divalent cations, particularly Ca^2+^, affect gel network formation and mechanical properties by promoting ionic interactions between proteins. In Ca^2+^-induced EW protein gels, increasing ion concentration has been associated with greater stiffness and elasticity; however, at higher concentrations, more extensive aggregation and coarser surface morphologies may occur [24,25]. Similarly, an adequate protein concentration is required to form a continuous three-dimensional network, and changes in protein concentration can affect network density, viscoelastic behavior, and final gel properties [22,24].

Overall, the biomaterial potential of EW arises from its high protein content, the coexistence of proteins bearing different functional groups, and the responsive behavior of these proteins to processing parameters such as pH, temperature, ionic environment, and concentration. These characteristics enable the formation of structures with distinct morphologies and physicochemical properties through controlled unfolding, aggregation, and crosslinking. Therefore, understanding EW-based biomaterial platforms such as hydrogels, nanoparticles, films, and three-dimensional scaffolds requires a holistic evaluation of protein composition, molecular interactions, and production conditions.

## 3. Production Methods of EW-Based Biomaterials

The applicability of EW in biomaterial production stems from its proteins’ ability to undergo conformational changes in response to physical, chemical, and biochemical stimuli and to assemble into diverse structures via intermolecular interactions. The unfolding, aggregation, and reorganization of globular proteins, particularly ovalbumin, can lead to continuous protein networks held together by hydrogen bonds, hydrophobic and electrostatic interactions, and sulfhydryl-disulfide exchanges [26]. However, in systems that rely solely on natural protein interactions, mechanical stability, degradation rate, and processability are often insufficient for many biomedical applications. Accordingly, several methods have been developed to convert EW into biomaterials, including physical gelation, chemical gelation, enzymatic crosslinking, chemical functionalization, and hybridization with other polymers or nanoscale components. The chosen production approach can directly influence network density, morphology, mechanical and rheological behavior, swelling and degradation properties, and ultimately biological performance. Some of the studies included in this section fall within the field of food science. These studies were utilized to elucidate the effect of processing conditions on the structural and mechanical properties of the polymeric network formed by EW proteins [27,28,29,30,31,32,33]. These data can guide biomaterial design; however, their biomedical significance requires validation at the biological level.

### 3.1. Physical Gelation

Physical gelation relies on the formation of three-dimensional network structures driven predominantly by non-covalent interactions among EW proteins and the rearrangement of their native reactive groups. It is one of the most fundamental production approaches because it does not require chemical crosslinking agents, is easy to apply, and leverages the natural gelling properties of EW. However, the properties of the resulting network are strongly influenced by parameters such as temperature, pH, ionic environment, and protein concentration during formation [22,34].

#### 3.1.1. Thermal Gelation

Thermal denaturation represents a well-established mechanism underlying the formation of three-dimensional network structures in EW proteins. During thermal processing, the native conformation of globular proteins is disrupted, thereby exposing hydrophobic amino acid residues in the interior and reactive sulfhydryl groups. These unfolding pathways enhance intermolecular interactions, specifically driving hydrophobic associations, hydrogen bonding, and sulfhydryl-disulfide exchanges, which cooperatively promote subsequent aggregation and matrix crosslinking. Crucially, the multi-component architecture of EW dictates the ultimate viscoelastic properties of the gel, governed by the specific fractionation and denaturation states of its constituent proteins. For instance, ref. [27] elucidated the foaming capacity and stability of EW proteins across diverse processing modalities, including ultraviolet irradiation, thermal treatment, high pressure, high-intensity ultrasound, and enzymatic hydrolysis. Their findings revealed that thermal treatment induces distinct protein aggregation, manifested by an increased particle size. Notably, while heating at 60 °C triggers the coagulation of ovotransferrin, escalating the temperature to 65 °C initiates the structural unfolding of ovalbumin, successfully liberating its latent sulfhydryl (-SH) groups. This demonstrates how the thermal gelation of EW proteins varies depending on the type of polysaccharide involved: neutral (konjac glucomannan), anionic (xanthan gum), or cationic (chitosan). This reveals that the charge of the polysaccharides directly governs the strength of protein–polysaccharide interactions, as well as the microstructure, textural properties, and water-holding capacity of the resulting gels [35].

In addition, the electrostatic and ionic environment established during thermal processing plays a pivotal role in modulating network topology. In a study highlighting the significant impact of base composition on thermal gelation [28], the effects of electrolyte addition on the denaturation and gelling characteristics of chicken EW proteins were comprehensively evaluated. Neutral salts, specifically sodium chloride, and alkaline variants (including phosphate- and carbonate-based salts) yielded divergent outcomes regarding gel firmness, microstructure, and color attributes. While NaCl deployment diminished gel firmness and durability alongside promoting larger aggregate sizes, alkaline salts elevated the net negative charge of the constituent proteins. This electrostatic repulsion culminated in the formation of matrices characterized by smaller, highly elastic aggregates with enhanced deformation tolerance [28]. Ultimately, these findings underscore that thermal gelation governs more than just temperature-dependent thresholds; it serves as a secondary mechanism to precisely modulate the mechanical and physicochemical architecture of the preformed protein network. Consequently, during biomaterial fabrication, thermal interventions do not merely induce phase solidification of the EW matrix. Rather, in synergy with environmental pH and ionic strength, they actively shape the final biomaterial by dictating its aggregate dimensions, network density, and macro-mechanical behavior.

#### 3.1.2. pH and Ionic Environment

These parameters serve as critical determinants in orchestrating the physical network assembly by modulating the net charge and subsequent aggregation kinetics of the constituent proteins. While enhanced electrostatic repulsion under conditions far from the isoelectric point facilitates controlled, ordered aggregation, a reduction in net charge induces the formation of coarser protein aggregates. Additionally, the specific impact of electrolyte systems extends beyond mere ionic strength, being highly sensitive to ion type and valence.

To leverage these fundamental parameters in a biomedical context, a 3D-printable hydrogel platform derived from fresh EW was recently engineered via a sequential crosslinking approach. In this system, initial physical gelation was induced using alkaline solutions (7.14–16.6 mg/mL NaOH) to promote carboxyl deprotonation and subsequent protein chain rearrangement, followed by the infiltration of cations (e.g., Na^+^ and Ca^2+^) to establish a secondary ionic network for mechanical tuning. This dual-network architecture not only facilitated direct 3D printing fabrication but also significantly enhanced fibroblast and adipose-derived stem cell proliferation and migration. Notably, these 3D-printed constructs upregulated angiogenesis and collagen deposition without requiring exogenous growth factors, effectively accelerating tissue repair in both normal and diabetic chronic wounds [36].

Utilizing a similar 3D printing approach, subsequent studies fabricated EW-based wound dressings characterized by an interconnected macroporous framework with pore sizes exceeding 100 μm [37]. This microstructural topography enhanced fibroblast viability and metabolic activity, which translated into accelerated wound closure, robust neovascularization, and organized collagen deposition in murine models. To further advance the mechanical and thermal boundaries of these matrices, a dual-crosslinked EW hydrogel was developed by combining alkaline NaOH treatment with a subsequent 5-day incubation in Dulbecco’s Modified Eagle Medium (DMEM) [38]. Incremental increases in Ca^2+^ concentrations within this system concurrently enhanced the hydrogel’s stiffness, thermal stability, and biocompatibility, despite a concomitant reduction in water-holding capacity. Given its improved cell-adhesive behavior in vitro, this dual-network formulation has been proposed as a potential candidate for bone tissue engineering and structural scaffolds.

#### 3.1.3. Ultrasound

Beyond chemical and ionic modifications, acoustic cavitation has emerged as an effective physical pretreatment strategy to modulate protein conformation and influence subsequent network assembly. For instance, in a food science study, ultrasound-assisted ethanol induction (utilizing 25–30% ethanol under 0–180 W sonication) was shown to be an effective approach to modify the textural and microstructural properties of egg white gels [29]. Optimizing the sonication power at 120 W yielded a notable enhancement in gel properties, resulting in an approximately twofold increase in firmness, a 1.5-fold increase in springiness, and a 1.3-fold improvement in water-holding capacity (WHC) relative to the untreated control group, with the highest gel performance consolidated at a 30% ethanol concentration [29].

The underlying structural transitions driving these macroscopic improvements are further elucidated by investigations into high-intensity ultrasound fields [30]. High-intensity sonication across varied power outputs systematically decreases surface tension, which subsequently amplifies protein solubility and enhances foaming capacity. At the molecular level, ultrasonic waves induce a more relaxed, open conformation in protein molecules while effectively depolymerizing the water-insoluble ovomucin network. This structural disassembly yields finer, highly dispersed aggregates and a highly interconnected porous matrix [30]. Although obtained in a food system, these findings suggest that ultrasound pretreatment may be used to tailor the microstructure of EW-based biomaterials.

#### 3.1.4. Freeze–Thaw

Alternative physical modalities, such as high hydrostatic pressure (HHP), have also been shown to effectively alter the conformational dynamics and aggregation kinetics of EW proteins [2]. However, within the field of biomaterial fabrication, the application of freeze–thaw processing remains predominantly confined to hybrid networks synthesized alongside crystallizable polymers, most notably poly(vinyl alcohol) (PVA) [39,40,41]. Furthermore, the investigation of high-pressure methodologies has historically been sequestered within food science applications rather than biomaterials engineering [4]. Consequently, these techniques should be conceptualized as auxiliary physical modulators capable of tuning protein conformation and subsequent network architecture, rather than as primary, standalone methods for the direct fabrication of EW-based biomaterials.

### 3.2. Chemical Gelation and Covalent Crosslinking

Chemical gelation involves the transition of a polymeric solution into a robust, three-dimensional macromolecular network mediated by the formation of permanent covalent bonds between constituent protein chains. Within EW matrices, native functional groups, specifically carboxyl, amino, and sulfhydryl moieties, serve as highly reactive sites for targeted chemical modifications. For instance, the synthesis of “polyalbumin” via the covalent crosslinking of primary amines in EW with diethylenetriamine (DETA) underscores the structural tunability of these systems. Lower DETA concentrations (≤5%) facilitate the fabrication of cohesive thin films, whereas elevated concentrations induce highly porous, foamy architectural morphologies [42]. The resulting biomaterial demonstrated a direct relationship between crosslink density and mechanical performance, exhibiting an elastic modulus of approximately 4 GPa, a tensile strength of 2 MPa, and ductility reaching 40%.

This chemical network also imparts distinct viscoelastic traits, including self-curing capabilities, high impact absorption, and crack-propagation resistance, while preserving good biocompatibility. Given its green-synthesis pathway and intrinsic biodegradability, this platform holds dual promise as a transient substrate for green electronics and as a matrix in biomedical fields [42].

To expand the application of chemical matrices toward advanced in vitro models, glutaraldehyde (GA) has been extensively utilized to develop active cryogel scaffolds from chicken EW for three-dimensional cell culture. Adjusting the GA-mediated crosslinking degree directly modulates the internal porosity and water-holding capacity of the cryogels [43]. When evaluating epithelial malignancies, such as HepG2 and MCF7 cancer lines, 3D configurations on these scaffolds yield higher biomimetic fidelity compared to conventional 2D cultures, presenting effective platforms for high-throughput drug screening and toxicological assays. Furthermore, GA-crosslinked EW scaffolds engineered with an elevated elastic modulus have been shown to upregulate the proliferation, migration, and multicellular spheroid aggregation of SKOV-3 ovarian cancer cells [43].

Collectively, the degree of chemical crosslinking emerges as a key governing parameter. While an increase in covalent bond density predictably elevates mechanical stiffness and structural stability, it concomitantly reduces network porosity, limits swelling ratios, suppresses molecular diffusion, and retards biodegradation rates. Consequently, chemical crosslinking in EW-based biomaterials is not limited to network stabilization; it also serves as a tool for controlling morphology, mass-transport profiles, and subsequent cellular interactions.

### 3.3. Enzymatic Crosslinking

Enzymatic crosslinking offers a compelling alternative to conventional chemical agents for modulating the structural properties of EW-based biomaterials, primarily because it facilitates the formation of site-selective covalent bonds under physiological or milder reaction conditions. Among various enzymatic systems, microbial transglutaminase (MTGase) has been extensively investigated due to its capacity to catalyze acyl transfer reactions. Specifically, MTGase establishes permanent protein–protein linkages by driving the formation of ε-(γ-glutamyl)-lysine isopeptide bonds between the γ-carboxamide groups of glutamine residues and the ε-amino groups of lysine residues [33].

However, the highly ordered, globular conformation of native EW proteins typically restricts the steric accessibility of these target glutamine and lysine residues to the enzyme’s active site. To overcome this limitation, thermal denaturation in an alkaline environment has been employed as an effective pretreatment strategy to render the proteins more susceptible to enzymatic recognition. Pretreating EW proteins at pH 11.3 and 85 °C for 30 min induces the formation of soluble thermal aggregates which, upon subsequent crosslinking with MTGase, exhibit significant improvements in key textural parameters, including gel strength, tensile strength, and deformation potential. The resulting matrix possessed a highly ordered and elastic network topology [31]. Although this system was not developed as a biomaterial, it may provide insight into the stabilization of heat-sensitive EW-based biomaterials.

To further optimize the microstructural landscapes of these matrices, recent strategies have integrated synergistic physical treatments and hybrid polymer blending. For instance, combining high-intensity ultrasound with MTGase-mediated crosslinking significantly enhances the physical and mechanical performance of EW–bovine gelatin hybrid hydrogels [32]. Here, ultrasonic cavitation partially unfolds the blended protein chains, exposing cryptic reactive sites to accelerate covalent bond density and yielding a significantly denser, less soluble gel network with programmable porosity. This dual-processing approach has been proposed for the encapsulation of heat-sensitive bioactive compounds, although it was evaluated only in food systems [32].

Beyond animal-derived matrices, MTGase has also been utilized to control network formation in sustainable, plant–animal hybrid biomaterials. When blending EW with hemp seed proteins under alkaline-heat and subsequent acid-induced gelation, the incorporation of the plant-based component typically compromises the elasticity and surface smoothness of the matrix. Nevertheless, the strategic introduction of MTGase-mediated crosslinking successfully compensates for this structural deficit by reinforcing the mechanical shear strength of the co-mingled network. This enzymatic intervention shows that the mechanical properties of EW–plant protein hybrid networks can be tuned, although their biocompatibility was not assessed [33].

### 3.4. Chemical Functionalization

Chemical functionalization operates on the principle of grafting novel reactive moieties onto protein backbones, distinct from the direct crosslinking of preexisting functional groups within EW matrices. This approach endows natural EW with advanced processability pathways, including photocrosslinkability, controlled radical polymerization, and compatibility with diverse biofabrication modalities. Within EW-based biomaterials engineering, the most prominent functionalization strategy involves the modification of proteins with methacryloyl or methacrylate groups [16,44].

A representative example of this approach is the development of an EW methacryloyl (EWMA) bioink designed for tissue engineering and bioprinting applications. By converting the native protein into a light-curable formulation, researchers engineered a highly bioactive hydrogel that effectively mimics the native extracellular matrix topology. In the presence of a photoinitiator, EWMA undergoes rapid covalent network formation under UV irradiation, wherein its rheological, mechanical, and degradation profiles can be precisely tuned by varying the hydrogel concentrations between 5%, 7.5%, and 10% (*w*/*v*). Both in vitro and in vivo evaluations have confirmed that this light-polymerized matrix supports cell proliferation, facilitates neovascularization, and exhibits good immunocompatibility without requiring elevated thermal processing [16].

The biomedical efficacy of the EWMA platform is particularly pronounced in 3D bioprinting and cell encapsulation applications. Photocrosslinkable EWMA bioinks can be readily fabricated into complex 3D geometries, maintaining high cell viability and proliferation post-encapsulation. Furthermore, human umbilical vein endothelial cells (HUVECs) successfully adhered to and proliferated across the EWMA topography; notably, a 7.5% (*w*/*v*) EWMA formulation induced significant cellular confluence and upregulated cadherin expression by day 7 of culture [16]. Consequently, methacrylate functionalization represents a highly efficient strategy to preserve the intrinsic bioactivity of EW while introducing light-driven network formation suitable for high-fidelity bioprinting.

To expand the mechanical and thermal boundaries of these photocurable systems, subsequent studies have explored the hybridization of EW with alternative structural proteins. For instance, light-crosslinkable hybrid bioinks (G-EW/SF) have been successfully synthesized by separately functionalizing EW and silk fibroin (SF) with glycidyl methacrylate (GMA) in the presence of the photoinitiator Irgacure 2959. Incorporating 50% G-SF substantially enhances the compressive modulus of the matrix and yields a well-defined three-dimensional porous framework with an average pore size of 61 (μm). Furthermore, the integration of SF shifts the thermal degradation threshold upward by approximately 10 °C relative to pure EW scaffolds, while concurrently retarding enzymatic degradation by retaining 18% more residual mass after nine days. Functionally, this composite architecture prolongs insulin release kinetics and markedly improves the adhesion and proliferation of L-929 fibroblasts [45].

Beyond methacrylation, the native -SH/disulfide exchange dynamics and polyphenol/polysaccharide conjugation pathways intrinsic to EW offer underexplored opportunities for designing multifunctional biomaterials [46]. Although previous studies have mainly applied these conjugation strategies in food technology and simple carrier systems [47], adapting these functionalization routes may provide new opportunities for tissue engineering. Overall, chemical functionalization extends beyond simple molecular alteration; it expands the processing options for EW proteins by introducing controllable reaction centers.

### 3.5. Hybridization with Other Polymers and Nanomaterials

Although the intrinsic bioactivity and versatile gelation kinetics of native EW offer distinct advantages for biomaterial fabrication, standalone EW matrices frequently suffer from subpar mechanical strength, poor long-term structural stability, and limited shape fidelity during 3D bioprinting processes [3,45]. To circumvent these rheological and structural bottlenecks, extensive research has targeted the formulation of hybrid networks. Integrating EW with functional polysaccharides, tailorable synthetic polymers, or engineered nanomaterials represents a potent strategy to synergistically reinforce its mechanical, rheological, and functional performance while preserving its foundational biological efficacy [39,48].

#### 3.5.1. Polymeric Hybrid Systems

Research involving bioinks composed of a mixture of sodium alginate (NaAlg) and egg white (albumin) represents one of the studies conducted on various natural macromolecules. While alginate effectively overcame the inherent low viscosity and suboptimal shape retention of natural EW, its capacity for divalent cation-mediated (Ca^2+^) ionic crosslinking ensured robust structural stability post-printing. In this context, scaffolds with suitable fluidity and viscosity were produced by combining EW optimized for 3D organ-printing applications with crosslinking mechanisms [48]. The resulting composite scaffold not only supported the adhesion and long-term viability of human umbilical vein endothelial cells (HUVECs) but also demonstrated the ability to promote the formation of new vascular networks (neovascularization) between the printed fibers. These results demonstrate that such composite scaffolds possess significant potential for tissue regeneration due to their biological functionality [48].

To further exploit this polysaccharide–protein synergy, photocrosslinkable or cell-laden architectural constructs have been fabricated using tailored natural bioinks. For instance, blending 1–3% (*w*/*v*) sodium alginate with pasteurized EW yielded improved extrusion printability, particularly within the 2–3% (*w*/*v*) alginate concentration range. The resulting bioprinted constructs exhibited an elastic modulus spanning 20–27 kPa and sustained a high encapsulated endothelial cell viability of 94% at day 7 post-fabrication. By successfully combining the shear-thinning printability of alginate with the structural durability and intrinsic bioactivity of EW, this system establishes a promising blueprint for advanced tissue constructs [49].

This structural design approach has been extended to ternary systems, such as alginate-methylcellulose (AlgMC) hydrogels, where the integration of EW serves to overcome the baseline shape-fidelity limitations of AlgMC while simultaneously amplifying cell–matrix interactions [48]. In these EW-augmented frameworks, the post-printing survival rate of primary human preosteoblasts reached a high value of 93.7 ± 0.15%. Furthermore, the protein-rich microenvironment actively facilitated cell spreading, migration, and the upregulated transcription of key osteogenic biomarkers, specifically bone sialoprotein (*IBSP*) and osteocalcin (*BGLAP*). These findings suggest that polysaccharide hybridization in EW systems is not limited to rheological modification but may also act as a biochemical cue that enhances the cell-instructive properties of the polymeric matrix [48].

Shifting the focus to synthetic polymeric networks, poly(vinyl alcohol) (PVA) has been frequently copolymerized with EW, particularly for the development of advanced wound dressings. Merging the high mechanical durability and film-forming characteristics of PVA with the bioactive, nutrient-dense profile of EW enables precise control over the matrix’s swelling kinetics, degradation rates, and biological responsiveness. For example, hybrid films synthesized with systematically varied PVA/EW ratios demonstrated that the compositional balance directly dictates the internal micromorphology, tensile profiles, and hydrolytic degradation behaviors of the dressings. When functionalized via encapsulation of nanoniosomal deferoxamine, these hybrid matrices robustly promoted human dermal fibroblast attachment and accelerated HUVEC lateral migration, highlighting their strong proangiogenic and regenerative efficacy in chronic wound healing paradigms [50].

Collectively, these systematic investigations demonstrate that polymeric hybridization within EW-based systems should not be narrowly conceptualized as a mere tool for mechanical reinforcement. Depending on the macromolecular architecture of the co-polymer, hybridization serves as a multidimensional regulatory mechanism capable of programming network topology, swelling capacities, shear printability, degradation profiles, and downstream cellular phenotypes.

#### 3.5.2. Hybrid Systems with Nanomaterials

The incorporation of nanomaterials into EW-based matrices enables the enhancement of their mechanical and physicochemical properties while introducing advanced functionalities, including electrical conductivity, controlled drug release, and antimicrobial activity. In a representative study, new nanocomposite hydrogels composed of EW, poly(vinyl alcohol) (PVA), and montmorillonite (MMT) nanoclay were engineered for potential use as wound dressings [39]. These hydrogels, fabricated via a cyclic freeze–thaw method with varied MMT concentrations (0, 5, and 10%), exhibited water-retention and moisture-transmission properties highly compatible with native biological tissues. The exfoliated MMT silicate layers functioned as ordered physical crosslinking nodes, thereby substantially enhancing the thermal stability and network resilience of the hydrogel. Furthermore, the resulting architectures provided optimal water vapor permeability to maintain a moist wound environment without inducing any cytotoxic effects [39]. In a subsequent in vivo validation, this EW/PVA/MMT system was shown to accelerate re-epithelialization relative to conventional gauze treatments while simultaneously improving the biomechanical properties of the regenerated tissue [40].

To expand the therapeutic repertoire of these bionanocomposites against infected wounds, recent studies have integrated broad-spectrum antibiotics within the nanoclay-reinforced frameworks. For instance, clindamycin-loaded bionanocomposite hydrogel dressings were synthesized via similar freeze–thaw processing of PVA and EW matrices. In these multi-component networks, the incorporation of functional nanoclays serves a dual purpose; it introduces additional structural reinforcement and establishes highly tortuous diffusion pathways that enable sustained, controlled drug release. Furthermore, increasing the nanoclay content systematically elevates the topographical roughness of the wound-contacting surface, thereby optimizing dressing adhesion and prolonged local retention of the therapeutic payload [41].

Beyond structural and barrier modifications, electrochemical functionality has been successfully introduced into EW-based matrices through the incorporation of carbon nanotubes (CNTs). By embedding conductive CNT networks within a physically crosslinked natural EW hydrogel, researchers engineered a platform that combines high stretchability, shear-thinning rheology, and intrinsic self-healing capabilities with high electrical conductivity. Leveraging the good extrusion printability of these formulations, 3D-printed EW-CNT flexible sensors were fabricated to detect subtle physiological and mechanical signals, including wrist pulses, complex finger articulations, and respiratory movements [51]. These breakthroughs demonstrate that carbon-based nanomaterial hybridization effectively extends the application scope of EW platforms beyond conventional tissue scaffolds into the domain of soft bioelectronics and wearable healthcare monitors.

In addition to carbon nanotubes, two-dimensional carbon derivatives like graphene oxide (GO) have demonstrated profound efficacy in modulating both the structural integrity and the biological responsiveness of these matrices. For example, an innovative electrospun wound dressing composed of EW proteins, PVA, and systematically varied concentrations of GO (0–2 mg/mL) was recently developed [52]. In the evaluation of wound dressings fabricated via electrospinning and composed of EW Protein, Polyvinyl Alcohol (PVA), and Graphene Oxide (GO), it was observed that dressings containing 1 mg/mL GO exhibited the most favorable hydrophilic, mechanical, and thermal properties among the tested formulations, thereby further enhancing tissue repair. The study demonstrated that these dressings promote the viability and proliferation of human dermal fibroblasts and endothelial cells while simultaneously providing effective antibacterial protection [52].

An overall evaluation of these studies indicates that nanomaterial hybridization provides an effective strategy for complementing the functional properties of EW-based matrices without fundamentally disrupting the native protein conformation. As reviewed herein, EW–alginate systems optimize shear printability and downstream cellular phenotypes, yielding soft constructs (20–27 kPa) with post-printing cell viabilities of 93.7–94% [48,49,53]; EW-PVA blends enable precise modulation of tensile strength and fluid dynamics; EW/PVA-MMT nanocomposites drastically improve network stability and controlled drug delivery [39,40,41]; and EW-CNT systems introduce advanced electrical properties for bioelectronic applications. However, increasing the amount of reinforcing filler does not necessarily improve performance; instead, an optimal concentration typically exists. For example, EW films containing 2 wt% chitin nanocrystals showed the highest tensile strength, whereas 5 wt% caused aggregation and embrittlement [54]. Similarly, among EW/PVA dressings containing 0–2 mg/mL graphene oxide, the best antibacterial and regenerative performance was obtained at 1 mg/mL [52]. Accordingly, the future design of EW hybrid systems must transcend simple mechanical reinforcement, focusing instead on the multidimensional optimization of complementary properties to achieve high-performance translational biomaterials.

## 4. Morphological Applications of EW-Based Biomaterials

The ability of EW proteins to form gels and films, stabilize foams, undergo molecular aggregation, and interact with various polymers enables this natural proteinaceous source to be engineered into biomaterials with diverse dimensions and architectures. Depending on the fabrication modalities, crosslinking strategies, and dehydration techniques employed, EW can be systematically processed into hydrogels, micro- and nanoparticles, continuous films, protective membranes, electrospun micro/nanofibers, biomimetic sponges, three-dimensional porous scaffolds, cryogels, and aerogels [3,9].

This morphological diversity determines not only the geometric configuration of the resulting construct but also fundamentally governs its specific surface area, pore size and interconnectivity, water absorption capacity, mass-transfer characteristics, mechanical performance, degradation kinetics, and downstream cell–material interactions. For example, highly hydrated hydrogel networks can provide microenvironments that resemble soft tissues, whereas biomimetic nanofibrous structures can successfully recapitulate the fibrillar topology of the native extracellular matrix [55]. Macroporous sponges, in turn, provide three-dimensional spaces that support host cell infiltration and vascularization [56]. Similarly, the high specific surface area and short diffusion distances of nanoparticulate systems can facilitate drug delivery, while the continuous and thin architecture of structured films offers distinct advantages in barrier and wound-dressing applications [50]. Therefore, when evaluating the biomedical performance of EW-based systems, the structural morphology must be considered a fundamental design parameter alongside their chemical formulation and crosslinking mechanisms (Table 1).

### 4.1. EW-Based Hydrogels

Hydrogels represent one of the most extensively investigated morphological forms of EW-based biomaterials for biomedical applications. The hydrophilic three-dimensional networks formed by EW proteins retain substantial amounts of water, thereby generating a hydrated microenvironment that partially mimics the native extracellular matrix (ECM) [2]. However, the biomedical performance of EW hydrogels depends not only on their high water content but also on the delicate balance among network density, pore size and interconnectivity, swelling kinetics, mechanical stability, and molecular transport. Systematic regulation of these parameters enables EW hydrogels to be tailored for a broad range of applications, including cell delivery [20], tissue engineering and three-dimensional (3D) bioprinting [16], wound healing [57], and localized therapeutic delivery [58].

The structural organization of the EW hydrogel matrix can be dynamically modulated through targeted ionic interactions. For instance, the secondary crosslinking of EW through the incorporation of Ca^2+^ ions following NaOH-induced gelation alters the protein secondary structure and aggregation behavior, resulting in more stable networks. Increasing CaCl_2_ concentrations modifies the swelling behavior and pore organization while markedly improving mechanical properties, including hardness, cohesiveness, and elasticity, which supports their potential application in bone tissue engineering [38]. To address soft tissue regeneration, such as chronic diabetic wounds, cost-effective and bioactive fresh EW frameworks can be engineered to balance structural integrity with biological responsiveness [36]. These dynamic formulations exhibit tailorable shear-thinning rheology suitable for extrusion-based 3D bioprinting into controlled microarchitectures, supporting the migration and proliferation of encapsulated fibroblasts and adipose-derived stem cells while promoting angiogenesis and collagen deposition in vivo [36].

The architectural design of 3D-printed EW hydrogels underscores the critical role of interconnected porosity in establishing pro-regenerative microenvironments. Incorporating defined microporous channels within physically crosslinked hydrogel frameworks accelerates cellular transport, enhances nutrient diffusion, and promotes robust neovascularization and tissue remodeling within wound models [37]. Beyond pristine protein matrices, hybridizing EW networks with alternative functional biopolymers offers a synergistic strategy to overcome baseline physical limitations. Co-polymerizing hydroxypropyl chitosan with EW under physical crosslinking yields multifunctional hydrogels engineered for infected burn wounds, combining anti-inflammatory pathways and reactive oxygen species (ROS) scavenging with antibacterial efficacy [57]. Similarly, blending EW with polysaccharides like κ-carrageenan enhances the long-term physicochemical durability and mechanical stability of the matrix [59]. This bio-hybrid approach can be further extended through the synthesis of binary EW–collagen networks or plant-derived polysaccharide-infused composite frameworks, which successfully modulate enzymatic degradation kinetics while preserving the foundational cytocompatibility of the matrix. This macromolecular versatility allows EW–gelatin hybrids to be processed across distinct morphological hierarchies, spanning from two-dimensional surface coatings to complex 3D microarchitectures within microfluidic systems [60]. Compared to inorganic materials such as silicon and glass, polymers are highly suitable and accessible materials that possess many promising application characteristics and can meet the personalized application requirements of disposable biomedical microfluidic devices [61].

Photocrosslinkable EW derivatives introduce precise spatial and temporal control over hydrogel formation. By functionalizing the protein backbone via methacryloyl modification (EWMA), the covalent crosslinking density can be regulated by adjusting the protein concentration and light exposure dose. This light-driven network formation bypasses the need for high temperatures, expanding the utility of EW hydrogels from bulk-molded constructs to cell-laden 3D bioprinted scaffolds and cellular encapsulation systems [16,62]. Furthermore, these crosslinked protein networks serve as bioactive matrices supporting cellular attachment and proliferation, which highlights their promise in functional scaffold design [17]. Optimizing the extrusion behavior, deposition fidelity, and print-quality parameters of photocurable bioinks formulated with GelMA and EW proteins establishes a reproducible biofabrication protocol for complex 3D constructs [63]. In parallel, reinforcing these hydrogels with nanoscale constituents represents a vital avenue to overcome the mechanical limitations of pure protein systems. Integrating functional nanocomposite fillers, such as silicate nanosheets and clay minerals, enhances mechanical stiffness and structural integrity for wound dressings and regenerative medicine [2,3]. Alternatively, the strategic incorporation of carbon dots (CDs) as biocompatible crosslinking agents offers a novel route to engineer resilient EW hydrogels with expanded pore networks that align their degradation rates with complex physiological regeneration cycles. Moreover, exploiting the responsive swelling and permeability of protein networks to variations in pH, temperature, and ionic strength enables EW hydrogel platforms to serve as smart matrices for the triggered release of therapeutics [63,64].

A higher degree of structural control can be achieved through the hierarchical organization of distinct pore architectures within a single construct. A biomimetic bilayer architecture can be engineered by exploiting the intrinsic foaming capacity of EW to combine a porous, dermis-like sublayer with a denser, Ca^2+^-stabilized, epidermis-like top layer. This multiscale macroporous scaffold promotes the directional migration of keratinocytes and fibroblasts, significantly accelerating wound healing in murine models [20]. At the molecular level, a cooperative combination of hydrogen bonding, hydrophobic interactions, and covalent linkages governs network formation in EW-based hydrogels, providing both structural flexibility and biodegradability. Structural stability and elasticity are further enhanced by the oxidative crosslinking of sulfhydryl groups exposed during thermal or chemical denaturation [22,46]. During thermal treatment, hydrophobic associations among unfolded protein chains drive initial aggregation, whereas the reformation of hydrogen bonds during subsequent cooling stabilizes the entrapped matrix, while electrostatic repulsion and ionic bridges directly influence network porosity and elastic modulus [22,46].

Consequently, this microstructural equilibrium dictates that controlled aggregation promotes fine, homogeneous microstructures, whereas rapid and uncoordinated precipitation produces heterogeneous networks with broader pore-size distributions. Although increased crosslinking density improves mechanical resilience, it inevitably reduces pore dimensions and water uptake, potentially limiting cell infiltration and macromolecular diffusion [65]. Taken together, these findings demonstrate that EW hydrogels constitute versatile biomaterial platforms that can be adapted into ionically reinforced, cell-laden, polymeric hybrid, photocrosslinkable, nanocomposite, and hierarchically organized systems. Accordingly, the primary objective of morphological engineering in EW hydrogels is to establish an optimal balance among porous architecture, swelling capacity, mechanical integrity, and functional bioactivity.

### 4.2. Three-Dimensional Porous Structures, Foams, and Sponges

The intrinsic foaming and gelation properties of EW offer substantial advantages for the fabrication of highly porous three-dimensional (3D) biomaterials. During interfacial aeration, the reorganization of amphiphilic proteins at the air–liquid interface generates a transient porous matrix. This metastable network can subsequently be converted into stable, sponge-like tissue scaffolds through coordinated crosslinking, freezing, and drying processes. Accordingly, EW-based foams and sponges can function as 3D cellular microenvironments that support cell attachment at the material surface and, when sufficient pore interconnectivity is achieved, facilitate cellular infiltration into the scaffold core [2].

Ovomucin, a high-molecular-weight glycoprotein in EW, plays a central role in the development of this porous morphology. Owing to its amphiphilic character, ovomucin rapidly adsorbs at the air–liquid interface, undergoes partial conformational rearrangement, and forms an elastic, cohesive protein film around entrapped air bubbles [13]. Its extensive disulfide networks and complex carbohydrate chains stabilize this interfacial layer and enhance the mechanical integrity of the developing foam [15,57]. Thus, interfacial foaming is not merely a transient physical process; when combined with controlled processing, it can provide the basis for scalable and biologically functional scaffolds for tissue engineering [13,14].

Freeze-drying and covalent crosslinking are the principal strategies used to transform transient foams into permanent 3D architectures. Foams produced by high-shear agitation of purified ovomucin or whole EW can be molded into porous, sponge-like matrices through controlled freezing followed by ice sublimation [13,56]. To mitigate dissolution and limited mechanical stability under aqueous conditions, ovomucin has been combined with auxiliary biopolymers, including gelatin, and covalently reinforced using carbodiimide chemistry involving EDC/NHS. This co-crosslinking approach improves bulk mechanical performance while enabling control over pore size and spatial organization. For example, the reported ovomucin/gelatin scaffold series exhibited a hierarchical pore structure in which primary macropores (100–200 µm) coexisted with interconnected micropores of approximately 20 µm [56]. The macropores provided permissive pathways for cell migration and neovascularization, whereas the micropores contributed to capillary fluid retention and nutrient diffusion. These findings indicate that scaffold performance is determined not only by overall porosity but also by pore-size distribution, wall thickness, and interconnectivity. Under alternative processing conditions, including confined nanopore dehydration and thermal annealing, egg proteins can also be reorganized into compact and mechanically robust membranes [13].

The biomedical utility of volumetric EW architectures derives from their ability to reproduce key features of physiological 3D microenvironments. Foams and sponges provide topographical cues that support the adhesion, spreading, and phenotypic maintenance of anchorage-dependent cells, including osteoblasts and dermal fibroblasts. In bone tissue engineering, the incorporation of bioceramic phases such as hydroxyapatite into EW matrices combines the organic protein network with bone-mimetic mineral components, producing bioactive composite scaffolds that may support osteogenic differentiation [13,14]. In soft-tissue regeneration, the high water-uptake capacity, interconnected porosity, and intrinsic bioactivity of EW proteins offer additional therapeutic potential. The extensive cellular infiltration and microvascular ingrowth reported for 3D ovomucin–gelatin constructs further support their regenerative relevance. Nevertheless, favorable immunological and angiogenic outcomes should not be generalized across all EW-derived porous matrices, as they depend on the specific protein fraction, crosslinking chemistry, scaffold architecture, and in vivo model under investigation [14,56].

The capacity of hierarchical morphology to regulate biological behavior is particularly evident in multilayered EW constructs. Whipping-induced EW foam was used to fabricate a porous, dermis-like sublayer coupled with a dense, Ca^2+^-stabilized, epidermis-like barrier. This biomimetic bilayer supported the spatial organization of fibroblasts and keratinocytes while reducing prolonged inflammation in a murine excisional wound model [19]. These findings demonstrate that morphology in EW-based systems contributes more than simply providing void space. Instead, tailored porosity, pore-size gradients, and structural hierarchy function as physical cues that guide cell distribution and promote tissue reorganization.

### 4.3. Nanoparticles and Microparticles

The compact globular architecture, amphiphilic balance, and intrinsic self-assembly propensity of EW proteins make them highly suitable macromolecular building blocks for colloidal micro- and nanoscale delivery systems. Ovalbumin, the predominant protein fraction in EW, is particularly attractive for particulate synthesis because of its high abundance and diverse array of reactive surface groups. EW protein (EWP) nanoparticles are commonly prepared through thermal aggregation, antisolvent precipitation, acid-heat treatment, or crosslinker-mediated condensation. These processes induce partial thermal or chemical unfolding, thereby exposing hydrophobic domains and promoting controlled self-association into discrete nanoscale cores. The hydrodynamic diameter, polydispersity index, and surface zeta potential of the resulting particles can be precisely modulated by adjusting the protein concentration, pH, ionic strength, temperature, and crosslinking kinetics [66].

A major application of EWP nanoparticles is the stabilization and delivery of labile bioactive compounds under physiologically challenging conditions. In one study, nisin-loaded EWP nanoparticles with an average size of approximately 112.5 nm were fabricated using acid-heat induction followed by ultrasonication [67]. The resulting system exhibited an encapsulation efficiency of 91.82% and high thermal stability. The proteinaceous shell protected nisin from proteolytic degradation while preserving its antibacterial activity, demonstrating that particulate encapsulation functions not only as a size-reduction strategy but also as an effective protective molecular barrier. In addition to serving as a carrier material, EW has been employed as a biogenic reducing and capping matrix for the green synthesis of zinc oxide nanoparticles ranging from 27.7 to 52.2 nm [68]. Copper nanoparticles and silver-doped copper nanoparticles were synthesized using the natural biopolymers gelatin and egg white (albumin). The anticancer effects of the synthesized bimetallic nanoparticles on liver cancer (HepG2) cell lines were investigated, and it was determined that both formulations reduced cell viability in a dose-dependent manner. The results of the tests demonstrated that optimizing the structural properties of these biocompatible nanomaterials could play a critical role in enhancing their efficacy in cancer treatment [69].

The high specific surface area of nanoscale carriers can substantially enhance their interaction with biological barriers, influence cellular uptake kinetics, and modulate cargo-release behavior. Nevertheless, particle size, surface charge, and colloidal stability must be carefully optimized, particularly in immunomodulatory and vaccine-delivery applications. In this regard, particulate systems containing egg-derived proteins have demonstrated considerable potential for transdermal immunotherapy. For instance, EW lysozyme encapsulated in chitosan-coated PLGA nanoparticles with an average size of approximately 100.7 nm and administered by iontophoresis achieved a 5.6-fold increase in transdermal antigen penetration and subsequent cellular uptake compared with the free antigen. This formulation also elicited a robust humoral immune response, characterized by markedly higher antibody titers than those induced by conventional needle-based injection [70].

The translational potential of EW-associated particulate platforms extends across a broad range of biomedical applications. Nanoconjugates incorporating curcumin and gold nanoparticles with particle sizes of approximately 200–300 nm have exhibited synergistic antibacterial and wound-healing activities, thereby offering multifunctional potential for regenerative medicine [71]. In neurodegenerative disease models, polyvinylpyrrolidone-modified gold nanoparticles reduced pathological lysozyme aggregation and amyloid fibril formation by approximately 60% [72]. Moreover, when incorporated into Pickering emulsions, EWP nanoparticles can enhance interfacial stability, gastrointestinal persistence, and the oral bioavailability of lipophilic nutraceuticals and therapeutic agents [73]. Collectively, these findings demonstrate that the complex molecular architecture and conformational responsiveness of EW proteins can be exploited to develop stimuli-responsive delivery vehicles for pharmaceutical, biomedical, and nutraceutical applications [74,75]. Studies exist that highlight the importance of hydrogel-based micro- and nanosystems in therapeutic and biomedical applications and address novel approaches used in drug delivery systems and cancer treatment. Research has been presented aiming to enhance the efficacy of these materials in the field of personalized medicine by optimizing their biocompatibility, mechanical stability, and stimulus-responsive properties (pH, temperature, light) [76].

Compared with nanoscale carriers, microparticulate EWP systems exhibit distinct pharmacokinetic and release characteristics owing to their larger particle diameters and lower surface-to-volume ratios. Their higher payload capacity and longer intramatrix diffusion pathways make them particularly attractive for sustained, localized drug release over extended periods. Although EW-based microparticles remain less extensively investigated than their nanoscale counterparts, they represent a promising and largely underexplored platform for depot-based therapeutic delivery and tissue-engineering applications.

### 4.4. Films and Membranes

The ability of EW proteins to assemble into continuous and cohesive networks during dehydration enables the facile fabrication of thin films and biological membranes [15,77]. Compared with highly hydrated hydrogel systems, these matrices exhibit a denser two-dimensional architecture and lower moisture content, rendering them particularly attractive for wound dressings, selective barrier layers, cell-culture substrates, and flexible biosensor interfaces [50,78].

The morphological and functional characteristics of EWP films are primarily governed by chain-packing kinetics during drying, plasticizer concentration, and the incorporation of secondary functional polymers [77,79]. Although pristine EWP films generally form homogeneous and optically transparent sheets, their intrinsic brittleness and pronounced moisture sensitivity remain major limitations [77,80]. Low-molecular-weight plasticizers, such as glycerol, enhance segmental chain mobility and consequently improve film flexibility. Conversely, the incorporation of hydrophobic domains can reduce water transmission and enhance moisture resistance. In addition, pristine EWP films exhibit good oxygen-barrier performance, particularly under low-relative-humidity conditions, which can be attributed to the compact spatial organization of the protein network and the extensive intermolecular interactions among globular protein domains [79].

From a biomedical perspective, blending EW with synthetic or natural polymers offers an effective strategy for overcoming its mechanical limitations while preserving its inherent bioactivity. Among these systems, poly(vinyl alcohol) (PVA)-EWP composite films have attracted considerable attention. PVA contributes mechanical strength, flexibility, and film-forming stability, whereas the EW component provides bioactive cues that may facilitate cell adhesion. For instance, PVA/EW composite films loaded with deferoxamine enhanced human umbilical vein endothelial cell (HUVEC) migration and in vitro tubule formation while supporting robust fibroblast proliferation. These findings highlight the potential of such composites as pro-angiogenic wound-dressing materials [50]. Similarly, highly transparent and pliable EW films reinforced with hydroxypropyl methylcellulose (HPMC) demonstrate that EWP-based matrices may extend beyond conventional wound-care applications and serve as flexible substrates for electronic skin and self-powered biomechanical sensors [78].

To further optimize the balance between mechanical strength and barrier performance, nanoscale reinforcements, including chitin nanocrystals (ChNCs), have been incorporated into EW matrices. Nanocomposite films containing 2 wt% ChNCs exhibited the highest tensile strength without compromising ductility, which was attributed to the uniform dispersion of the nanofillers and efficient interfacial stress transfer. In contrast, increasing the ChNC content to 5 wt% promoted particle agglomeration, approximately doubled the matrix stiffness at the expense of tensile strain, and induced premature embrittlement [54]. These findings emphasize the importance of controlling nanofiller concentration to achieve an optimal combination of strength, extensibility, and structural integrity.

In addition, superabsorbent hybrid hydrofilms have been developed by combining EW with Carbomer 940 to address the exudate-management challenges associated with chronic wounds [81]. The incorporation of Carbomer 940 enhanced polymer-chain packing and improved structural integrity, resulting in water-holding capacities of up to 1005%, sustained protein-release profiles, and modulation of the water-vapor transmission rate. These biocompatible hydrofilms also promoted fibroblast proliferation and directed cell migration, thereby providing a promising biomaterial platform for impaired healing and poor vascularization in diabetic foot ulcers [81]. The conversion of waste egg white proteins and chitosan (derived from the food industry) into polyacrylonitrile nanofiber membranes via electrospinning has been investigated. Tests have demonstrated that these eco-friendly materials exhibit high efficacy, particularly against bacteria such as *Escherichia coli* and *Staphylococcus aureus.* Furthermore, the reusability and storage stability displayed by this material indicate its potential for application in fields such as biomedicine and food packaging [82].

Collectively, EW-based films and membranes offer high surface-to-volume ratios and may be adapted for clinical applications, pending further in vivo and clinical validation. Nevertheless, cellular infiltration within these materials is inherently limited because cell penetration occurs predominantly from their planar surfaces. Accordingly, rather than serving as volumetric scaffolds for deep-tissue regeneration, EW-based films are more appropriately positioned as barrier membranes, topical wound dressings, bioactive coatings, and bio-interfacial sensing platforms.

### 4.5. Fibers and Nanofibers

Micro- and nanoscale fibrous architectures constitute a particularly promising class of EW-based biomaterials for wound healing and regenerative medicine. Their high surface-area-to-volume ratio, interconnected porous structure, and close architectural resemblance to the fibrillar collagen networks of the native extracellular matrix (ECM) provide an advantageous structural framework for cell–material interactions. Fiber diameter, alignment, inter-fiber spacing, and membrane porosity collectively regulate cellular adhesion, polarization, and migration, while also governing gas exchange and the transport of therapeutic agents [47,83]. Accordingly, electrospun EW matrices function not merely as passive physical supports but as dynamic topographical microenvironments that actively influence cellular behavior and tissue regeneration.

However, the fabrication of uniform and continuous fibers from pristine EW by electrospinning remains technically challenging. The globular quaternary structures of the major EW proteins, together with their limited chain entanglement in solution, hinder the formation of a stable polymer jet during electrostatic stretching. To overcome these rheological limitations, EW is commonly co-dissolved with synthetic or natural carrier polymers capable of forming continuous fibers, including poly(ethylene oxide) (PEO), poly(vinyl alcohol) (PVA), polycaprolactone (PCL), and gelatin [47,84]. These auxiliary polymers provide the viscoelasticity and molecular entanglement required for stable jet elongation while preserving the biochemical functionalities of the dispersed EW phase. Subsequent covalent crosslinking, typically achieved through carbodiimide-mediated chemistry, such as the use of 1-ethyl-3-(3-dimethylaminopropyl)carbodiimide (EDC), or through thermal stabilization, is essential for preventing spontaneous dissolution under aqueous conditions and for modulating the degradation kinetics of the resulting matrices [14,50].

This approach has been successfully demonstrated in EW/PVA electrospun wound-dressing platforms. The resulting open, nonwoven nanofibrous mesh provides a physical barrier against exogenous microbial invasion while maintaining high moisture-vapor transmission and facilitating cellular infiltration. Building on this design, an asymmetric bilayer EW/PVA nanofibrous scaffold incorporating deferoxamine was developed to combine biomimetic topography with controlled therapeutic delivery. The scaffold sustained deferoxamine release for approximately 9 days and elicited pronounced angiogenic responses in both in vitro and ex vivo models. In a full-thickness cutaneous excision model in rats, the composite mesh promoted early neovascularization, accelerated wound closure, and resulted in complete re-epithelialization within 10 days [14,50].

Taken together, these findings demonstrate that EW-based nanofibers can integrate two critical functionalities within a single morphological platform. Their fibrillar, tissue-scaffold-like topography supports cell–material interactions, whereas their high specific surface area provides an effective substrate for the loading and controlled release of therapeutic agents. Nevertheless, the intrinsically hydrophilic proteinaceous structure of EW remains a notable limitation, as it may induce excessive fiber swelling under aqueous conditions and compromise the morphological integrity of the fibrous matrix.

### 4.6. Cryogels and Aerogels

Cryogels are macroporous three-dimensional biomaterials produced through cryotropic gelation, a process in which growing ice crystals function as temporary, sacrificial porogens. During subzero cooling, ice formation concentrates proteins and gel-forming constituents within a confined, unfrozen liquid microphase through cryoconcentration. Within this concentrated phase, molecular crosslinking and polymer-chain consolidation occur around the advancing ice crystals. Subsequent thawing removes the ice templates and generates an interconnected macroporous polymeric network. In comparison with conventionally fabricated dense hydrogels, cryogels exhibit substantially larger pore openings, typically ranging from several tens to several hundreds of micrometers, together with extensive pore interconnectivity. These features facilitate rapid convection-mediated fluid transport, efficient nutrient exchange, and deep cellular infiltration throughout the scaffold [85].

Because of its intrinsic capacity for both thermal and chemical gelation, whole EW represents an economical and bioactive macromolecular precursor for the fabrication of macroporous scaffolds. Early studies demonstrated that EW protein-based cryogels provide interconnected three-dimensional microenvironments capable of supporting cell adhesion, spreading, and sustained proliferation in vitro [43]. In addition, the rapid sponge-like rehydration behavior and high water-retention capacity of EW cryogels, combined with their inherent cytocompatibility, support their potential application in tissue engineering and three-dimensional in vitro culture systems [2,43].

Nevertheless, the translational potential of EW cryogels is determined by the complex interplay among architectural and mechanical parameters, including pore morphology, wall thickness, porosity, and bulk elasticity. Since globular egg proteins form particulate rather than fibrillar network backbones, unreinforced EW cryogels often display limited mechanical toughness and may undergo plastic deformation or structural collapse under repetitive cyclic loading [2,86]. Moreover, ice-templating is highly sensitive to thermal gradients, cooling rates, and solute concentrations. Consequently, the generation of uniform and reproducible pore architectures, particularly across large-volume constructs, remains a significant engineering challenge [2,46]. To address these limitations, the incorporation of structural biopolymers or nanofillers, together with the use of effective chemical or enzymatic crosslinking strategies, has emerged as a promising approach for improving scaffold stability and mechanical performance [3].

Aerogels, in contrast, constitute a distinct class of ultralight porous solids generated by removing the solvent from a gel precursor while minimizing the capillary forces that would otherwise cause network collapse. This is typically achieved through supercritical drying or carefully controlled freeze-drying, resulting in highly integrated, ultralow-density frameworks with very high internal specific surface areas. These characteristics render aerogels attractive for molecular entrapment, heterogeneous catalysis, and pulmonary or mucosal drug-delivery applications [87]. However, systematic investigations of unmodified EW-based aerogels for biomedical applications remain limited. Accordingly, aerogels should currently be regarded as an emerging and prospective research direction rather than an established biomaterial platform within the EW literature.

Overall, cryogelation and aerogel synthesis provide promising strategies for converting raw EW into highly interconnected three-dimensional macro- and mesoporous constructs [2,43]. Future studies should prioritize the improvement of architectural reproducibility, the enhancement of wet-state mechanical performance, and comprehensive in vivo validation. Such efforts will be essential for translating EW-derived porous platforms from proof-of-concept materials into clinically relevant scaffolds for tissue regeneration.

**Table 1:** EW-based morphological applications.

Biomaterials Composition	Biomaterials Type	Methods	Improvement	References
Ovomucin/gelatin (OVN)	Scaffold	Crosslinking	Porous scaffolds were crosslinked with EDC to obtain stable scaffolds with long-term degradation. The scaffolds with the highest concentration of OVN (OVN-75) showed micropores with an average diameter of 20 µm, as well as macropores of 100–200 µm in diameter	[56], (2017)
Egg white	Hydrogel Wound Dressing	Crosslinking	By increasing the expression of positive chemokines and growth factors in cells, it shortened the inflammatory response and improved wound healing. Transcriptome sequencing revealed significant upregulation in genes related to the cell cycle and metabolism.	[19], (2026)
Egg white	3D Hydrogel Scaffolds 2D Hydrocolloid Films	By sequential temperature-dependent gelation followed by chemical crosslinking	The large range of properties and characteristics of these materials provides a strong potential for a large variety of (regenerative medicine) TERM applications, including cancer models, organoid growth, compatibility with bioprinting, or implantable devices.	[60], (2023)
Nisin-loaded EWP nanoparticles	Gel	Nano-encapsulation	Thermal induction could improve the solubility, heat resistance, and encapsulation of nisin in the samples. In terms of antimicrobial function, acid-heat induction did not reduce the antimicrobial activity of nisin encapsulated in EW protein. Compared with free nisin, the loss rate of bactericidal activity of AH-NEn was reduced by 75.0% and 14.0% following treatment with trypsin or a thermal treatment at 90 °C for 30 min, respectively.	[67], (2024)
Hen egg-white lysozyme (HEL) (PLGA) chitosan hydroxypropyltrimonium chloride	Polymer Coating	Iontophoresis drug delivery	These positively charged carriers, combined with iontophoresis, significantly increased the penetration of the antigen into the skin and its absorption by cells. Experimental results demonstrated that this method generated a stronger immune response by producing more antibodies compared to traditional injections.	[70], (2021)
Chitin nanocrystals (ChNCs, 1–5 wt.%), EW protein (EWP), glycerin (GLY)	Film	Compression molding	Films containing 2% ChNC showed the highest tensile strength without losing their flexibility, thanks to homogeneous distribution. Adding 5% powdered ChNC made the films even harder; their flexibility decreased by half, and they became brittle.	[54], (2025)
Lyophilized powder of chicken EW, Carbomer-940 powder, glutaraldehyde, sodium chloride, potassium chloride, sodium phosphate dibasic, MTT	Hydrofilm	Chemically crosslinked	The cell viability assay demonstrates no toxicity, as well as high human foreskin fibroblast cell proliferation for the hybrid hydrofilm sample, where cell migration is positively affected in the presence of the bioactive components extracted from the dressing. Taken together, the optimized hybrid hydrofilm could be suggested as a promising wound dressing for DFU (Diabetic foot ulcer).	[81], (2024)
Bio-active EW, (0.25%, 0.5%, 0.75%, and 1%) of glutaraldehyde solution	Cryogel Scaffolds	Crosslink cryogelation	Given the distinct advantages of the bioscaffold derived from EW, such as homogeneous pore size, amide bonds during crosslinking, and a high degree of porosity, it has been shown that it can be effectively used in 3D culture. The successful development of chicken EW as a cost-effective cryogel scaffold for 3D cell culture for use in toxicology and pharmacology demonstrates its suitability for applications in these fields.	[43], (2019)

## 5. Biomedical Applications

EW-based biomaterials have attracted interest in a wide range of biomedical applications due to their natural biocompatibility, biodegradability, macromolecular richness, low cost, and structural versatility. In recent years, significant research has been conducted on polymeric biomaterials synthesized via chemical processes—including hydrogels, scaffolds, films, bioinks, and wound dressings—across a wide range of application areas [2,13,88] (Table 2).

The principal constituents of EW, including ovalbumin, ovotransferrin, ovomucin, and lysozyme, enable it to function not only as a passive structural scaffold but also as a bioactive platform capable of influencing cellular behavior and modulating the surrounding microenvironment [2]. Through appropriate processing strategies, EW can be engineered into hydrogels, films, electrospun nanofibers, colloidal particles, macroporous sponges, cryogels, and three-dimensional bioprinted constructs [13]. This versatility allows a single renewable protein source to be tailored to diverse clinical requirements, ranging from tissue regeneration to controlled drug delivery.

Furthermore, it has been noted that biopolymers—including polysaccharides and proteins—are potential candidates for optical biomaterials due to properties such as biocompatibility (stemming from their natural occurrence and recognition by the immune system), biodegradability, and sustainability [89].

However, the biological and mechanical performance of EW-based biomaterials is not determined solely by their protein composition. Key material parameters, including protein concentration, crosslinking chemistry and density, pore architecture, degradation kinetics, and interfacial integration with synthetic polymers or nanofillers, play a critical role in determining host responses, cell–matrix interactions, and overall therapeutic performance [14,86]. Accordingly, the rational design of EW-based biomaterials requires the coordinated optimization of both their intrinsic biochemical properties and their physicochemical architecture.

### 5.1. Tissue Engineering, Regenerative Medicine, and 3D Cell Culture

Scaffolds developed for tissue engineering must provide biomimetic structural support to promote cell adhesion and preserve cellular phenotype while simultaneously enabling efficient transport of oxygen, nutrients, and metabolic waste. Owing to its high water-retention capacity and versatile crosslinking chemistry, pristine and modified EW can be processed into hydrogels, cryogels, and sponge-like architectures suitable for the culture of various primary cell types, including dermal fibroblasts, vascular endothelial cells, chondrocytes, and multipotent mesenchymal stem cells (MSCs) [16,17].

Macroporous EW-based architectures are particularly promising because their open and interconnected pore networks facilitate deep cellular infiltration and neovascularization beyond the limitations of superficial cell seeding. EW-derived porous sponges have been shown to support the viability, metabolic activity, and proliferation of human dermal fibroblasts in vitro, while also promoting rapid cellular ingress and capillary integration following subcutaneous implantation. In addition, the intrinsic affinity of these scaffolds for vascular endothelial growth factor (VEGF) may support the formation of localized pro-angiogenic microenvironments [2]. Similarly, hierarchical ovomucin/gelatin scaffolds with bimodal pore distributions, consisting of primary macropores measuring 100–200 µm and interconnected micropores of approximately 20 µm, provide a favorable framework for cellular migration, fluid exchange, and tissue ingrowth [57]. In lyophilized EW sponges developed for cartilage repair, adjustment of the protein-to-solvent ratio enables controlled modulation of pore size and architecture. Such control facilitates the deposition of glycosaminoglycans and cartilage-like extracellular matrix (ECM) by articular chondrocytes [12].

**Figure 2:**
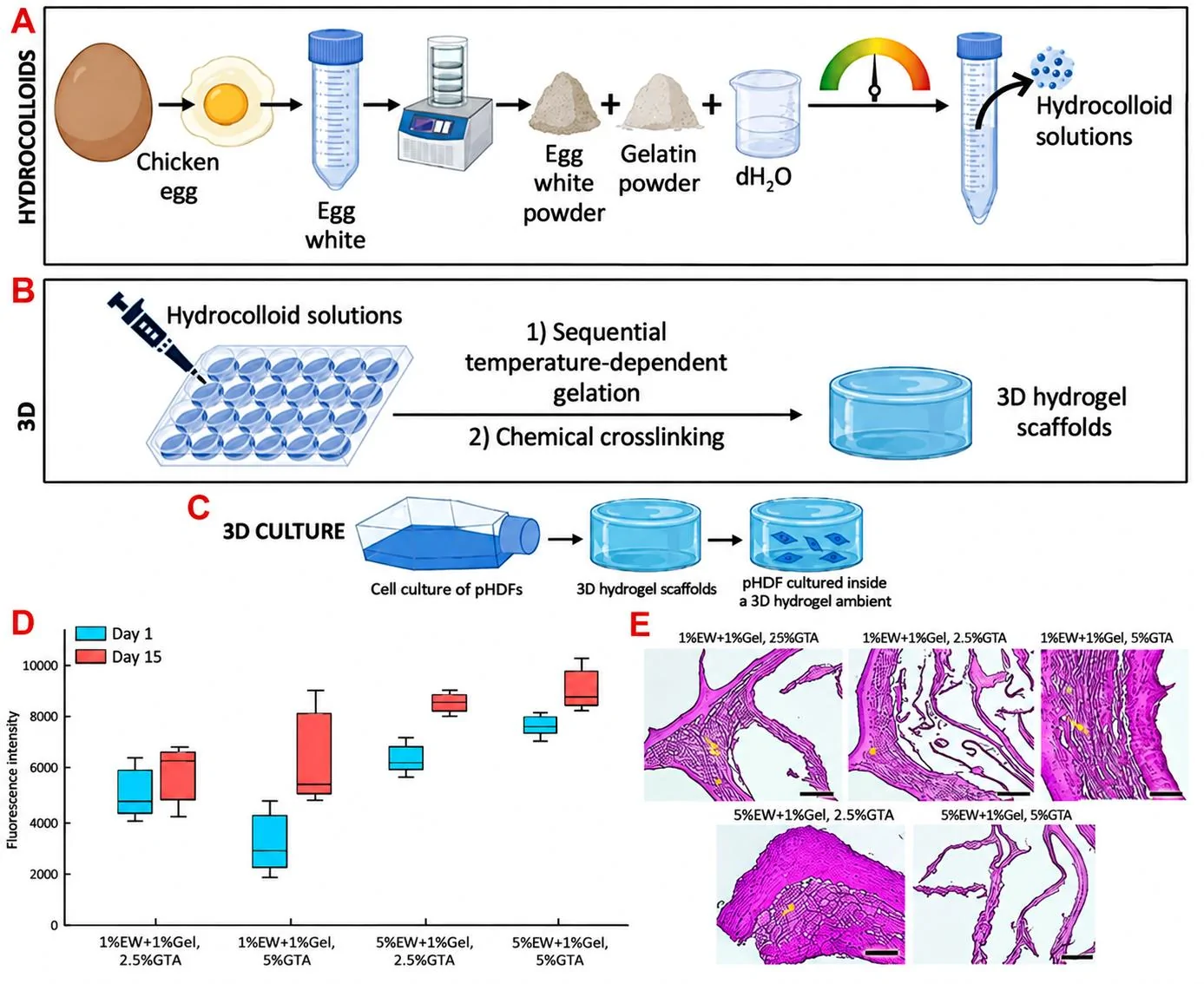
Representative application of egg white EW-based hydrogels in tissue engineering. (**A**) Preparation of EW/gelatin hydrocolloid solutions. (**B**) Fabrication of glutaraldehyde-crosslinked 3D hydrogel scaffolds. (**C**) Schematic representation of primary normal human dermal fibroblast (pnHDF) culture within the scaffolds. (**D**) Cell metabolic activity evaluated by the alamarBlue^®^ assay during culture. (**E**) Representative H&E-stained sections showing cellular distribution within the 3D hydrogel scaffolds after 10 days of culture. Scale bar = 50 µm Adapted from [60] with permission.

For three-dimensional in vitro cell culture, EW-derived hydrogels provide a physiologically relevant, protein-rich microenvironment that can promote spontaneous cellular organization and multicellular spheroid formation. Various cell lineages, including invasive breast carcinoma cells and salivary gland acinar cells, have exhibited physiologically relevant morphology and preserved phenotypic characteristics within EW-based matrices [90]. Moreover, the optical transparency of crosslinked EW hydrogels enables direct and non-invasive optical or confocal imaging of cellular migration and three-dimensional morphogenetic processes [60] (Figure 2). When combined with complementary polymers such as alginate, gelatin, or methylcellulose, EW can be formulated into shear-thinning composite bioinks with tunable mechanical properties, swelling behavior, and microfluidic printability [14,48,53,60]. These cell-laden hybrid constructs have demonstrated enhanced metabolic activity in endothelial cells and MSCs, highlighting EW as a cost-effective and chemically adaptable alternative to batch-variable, tumor-derived animal matrices such as Matrigel for disease modeling and high-throughput drug screening [4,12,16,18].

Despite these advantages, the inherent batch-to-batch variability of raw EW, together with the inverse relationship between scaffold porosity and compressive strength, necessitates careful optimization of EW-based systems. The development of reproducible sterilization protocols that preserve relevant conformational epitopes is particularly important. In addition, comprehensive long-term studies assessing phenotypic stability and multilineage differentiation will be required before EW-based scaffolds can be considered a broadly applicable substitute for established basement membrane extracts.

### 5.2. Drug and Bioactive Compound Delivery

The amphiphilic amino acid composition of EW proteins, characterized by discrete hydrophobic domains and charged peripheral residues, facilitates robust supramolecular interactions with therapeutic compounds spanning a broad range of physicochemical properties. Among these proteins, ovalbumin has emerged as an effective macromolecular depot for the encapsulation, stabilization, and controlled release of hydrophobic small molecules, peptides, and plant-derived bioactive compounds [4,74]. The fabrication of EW-based nanoparticles, microparticulate carriers, and swellable hydrogels not only helps preserve the structural integrity of encapsulated molecules against physiological degradation but also enables precise modulation of their spatiotemporal release profiles [13].

Carrier geometry and matrix crosslinking density are key determinants of drug-release behavior and, consequently, pharmacokinetic performance. In bulk hydrogel systems, cargo release generally occurs through the combined effects of network relaxation and swelling, passive diffusion through the hydrated matrix, and hydrolytic or proteolytic degradation of the polymeric network. By contrast, particulate carriers are governed primarily by parameters such as surface charge, hydrodynamic diameter, surface-area-to-volume ratio, and the density of the proteinaceous core. Moreover, the conformational responsiveness of protein backbones to changes in the local microenvironment, including pH, ionic strength, and enzymatic activity, provides a basis for designing intelligent and stimuli-responsive drug delivery systems [13,64].

For instance, 5-fluorouracil-loaded EW–chitosan nanoparticles, with particle sizes ranging from 530 to 850 nm, exhibited pronounced pH-dependent release behavior, with accelerated drug liberation under mildly acidic conditions (pH 5.0–6.0), which are characteristic of the tumor microenvironment, compared with the physiological pH of 7.4 [13]. Similarly, in oscillation systems produced using droplet microfluidics, studies on continuous fluid processing, particle formation, and consequently diffusion-mediated oscillation kinetics are also available.

In inorganic–organic bionanocomposite systems, freeze–thawed poly(vinyl alcohol)/EW hydrogels reinforced with montmorillonite clay demonstrated that increasing the MMT content reduced hydrogel swelling and substantially delayed amoxicillin diffusion. This retardation was attributed to the development of more tortuous diffusion pathways and enhanced electrostatic crosslinking. Under alkaline conditions, however, drug release was accelerated because of increased charge repulsion and greater hydrogel swelling [58].

Furthermore, the incorporation of curcumin-loaded niosomal nanovesicles into ultrasound-treated, transglutaminase-crosslinked EW–gelatin hydrogels produced dense, low-porosity networks with enhanced storage moduli (G′) and controlled biphasic release profiles [91]. Collectively, these findings indicate that the therapeutic performance of EW-based delivery systems cannot be evaluated solely on the basis of encapsulation efficiency. Instead, loading capacity, suppression of the initial burst release, stability under physiological conditions, and preservation of receptor-mediated bioactivity should be assessed concurrently.

### 5.3. Wound Healing and Exudate Management

Cutaneous wound healing is the most extensively validated biomedical application of EW-based biomaterials. The processing of EW into injectable hydrogels, transparent films, electrospun meshes, and macroporous sponges enables the integration of several critical wound-management functions within a single platform, including exudate absorption, physical barrier formation, cellular recruitment, and sustained drug delivery [2,17]. Although lysozyme provides a degree of intrinsic antibacterial activity, particularly against Gram-positive bacteria, native EW alone offers limited broad-spectrum antimicrobial protection. Accordingly, the incorporation of chitosan, metallic nanoparticles, or exogenous antibiotics into the EW protein matrix has substantially improved its therapeutic and translational potential.

One notable example is a physically crosslinked hydroxypropyl chitosan/EW hydrogel developed for the treatment of infected thermal burns [57]. This hybrid hydrogel accelerated re-epithelialization through multiple complementary mechanisms, including the neutralization of reactive oxygen species (ROS), attenuation of pro-inflammatory cytokine signaling, inhibition of bacterial colonization, and stimulation of local neovascularization. Likewise, topical matrices containing enzymatically derived EW hydrolysates and bioactive peptides achieved wound-closure rates exceeding 90% within 13 days in preclinical models [92].

Recent advances in EW-based wound dressings have also addressed the persistent hypoxic microenvironment characteristic of chronic, non-healing wounds. Photosynthetically active EW hydrogel microspheres encapsulating live Chlorella pyrenoidosa enabled continuous and localized oxygen generation at diabetic excision sites. This oxygen-producing system rapidly alleviated tissue hypoxia, promoted collagen-fiber realignment, and enhanced capillary ingrowth [93]. In related cell-therapy strategies, the encapsulation of adipose-derived stem cells (ADSCs) within injectable EW hydrogel microspheres improved cell retention and sustained both cellular viability and paracrine-factor secretion within the wound bed [20]. Moreover, radially oriented EW hydrogels loaded with lyophilized adipose tissue-derived extracellular vesicles (L-EVs) enabled the sustained and directionally controlled release of vesicular signals. This spatially regulated delivery promoted the transition of host macrophages from a persistent pro-inflammatory M1 phenotype toward a regenerative and pro-resolving M2 phenotype [21].

Hierarchical structural organization represents another important determinant of tissue repair. Biomimetic bilayer EW dressings fabricated through interfacial foaming were designed to reproduce key features of native skin architecture. The loosely porous dermal layer facilitated fibroblast infiltration, whereas the dense, Ca^2+^-annealed epidermal layer functioned as a microbial barrier and reduced transepidermal water loss. Together, these features contributed to a shorter inflammatory phase in murine wound models [20]. For highly exudative wounds, superabsorbent EW/Carbomer-940 hydrofilms exhibited a high swelling capacity of up to 1005% while maintaining dimensional integrity. In addition to limiting periwound maceration, these hydrofilms supported directional fibroblast migration and thereby contributed to a more favorable reparative microenvironment [81].

Ultimately, EW-based wound-healing technologies have evolved beyond their initial role as passive wound coverings. They now comprise multifunctional and increasingly responsive biomaterial platforms capable of simultaneously addressing chronic inflammation, tissue hypoxia, microbial infection, excessive exudate, and impaired tissue remodeling.

### 5.4. Biosensors and Bioelectronic Applications

The conformational stability, biocompatible surface chemistry, and abundance of functional anchoring sites in EW proteins render them attractive platforms for biosensing, bioelectrochemical applications, and flexible electronic devices [78,94]. In these systems, EW primarily serves as a protective matrix for enzyme immobilization [94], a viscoelastic structural binder for conductive nanomaterials, or an organic scaffold for the fabrication of functional micro- and nanostructures [95].

**Figure 3:**
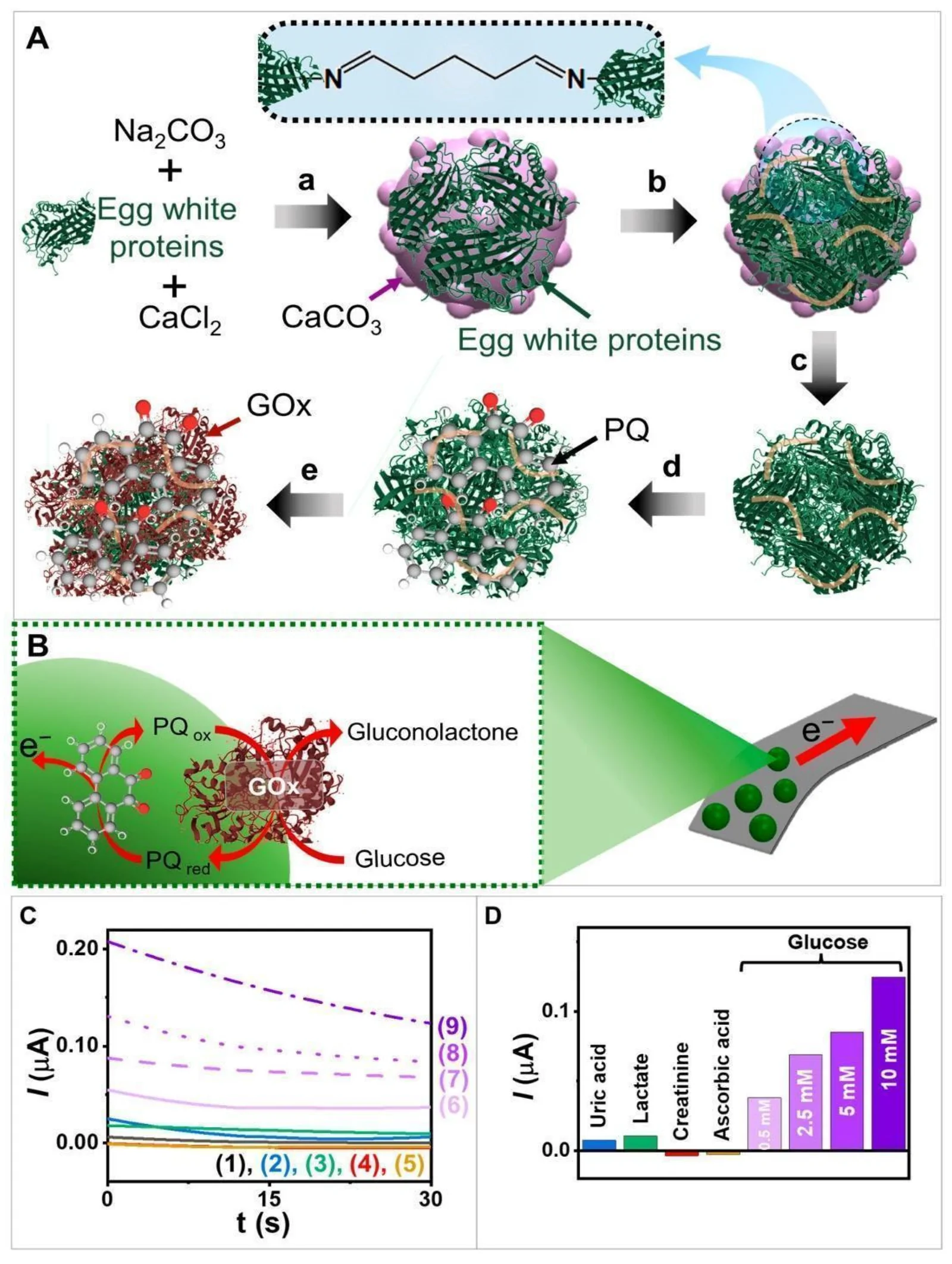
Egg white protein-based microparticles as a representative platform for electrochemical glucose biosensing. (**A**) Preparation and functionalization of egg white protein microparticles (MPs) with the redox mediator phenazine quinone (PQ) and glucose oxidase (GOx). (**B**) Schematic representation of the GOx/PQ/EW protein MP-modified electrode and mediated electron transfer during glucose oxidation. (**C**) Electrochemical responses to glucose and potential interfering compounds. (**D**) Comparison of sensor responses demonstrating preferential detection of glucose. Adapted from [96] with permission.

In enzymatic biosensing applications, ovalbumin-rich EW matrices provide a stabilizing, native-like aqueous microenvironment for labile biocatalysts, including glucose oxidase (GOx), while maintaining efficient substrate diffusion. In EW-based bioelectrodes functionalized with gold nanoparticles and carbon nanotubes, the protein matrix promoted rapid heterogeneous electron transfer through redox mediators while protecting the entrapped enzymes from proteolytic degradation and surface fouling. These electrodes retained more than 85% of their initial current response after 6 h of continuous operation. Moreover, the thickness of the protein layer served as an effective kinetic parameter for modulating both the dynamic linear range and the response time of the biosensor [94,96] (Figure 3). Beyond passive enzyme entrapment, EW proteins can also participate in the coordination of transition-metal ions. For example, hybrid organic–inorganic nanoflowers synthesized from EW proteins and copper phosphate functioned as catalytic signal amplifiers for the colorimetric detection of carcinoembryonic antigen (CEA), yielding a limit of detection as low as 3.52 pg/mL [95].

In flexible and wearable bioelectronic systems, the incorporation of carbon nanotubes (CNTs) or conducting polymers into plasticized EW films produces elastomeric conductive composites capable of real-time piezoresistive sensing. Mechanical deformation alters the internal conductive percolation network, resulting in measurable changes in electrical resistance. This mechanism enables the continuous monitoring of joint movement, arterial pulse waves, and respiratory activity. In addition, highly transparent EW films modified with hydroxypropyl methylcellulose (HPMC) demonstrate the potential of renewable protein-based matrices as flexible substrates for triboelectric nanogenerators and self-powered epidermal sensors [78].

Importantly, classifications in the literature should clearly distinguish studies in which EW functions as an active structural or sensing matrix from analytical investigations that consider endogenous EW proteins, such as lysozyme or ovomucoid, solely as diagnostic target analytes in allergen-detection platforms [97,98]. This distinction is essential for accurately evaluating the functional contribution of EW-derived proteins to biosensor design, bioelectrochemical performance, and flexible electronic applications.

**Table 2:** EW-based biomedical applications.

Biomaterials Composition	Biomaterials Type	Methods	Improvement	References
Chicken egg white, copper phosphate	Biosensor	One-Pot	The resulting colorimetric sensor has been successfully applied for the detection of CEA in human serum samples.	[95], (2021)
(AuNPs) and (Fc(COOH)2) on a glass carbon electrode (GCE)	Biosensor	DPV (For Electrochemical Sensor)	In the standard addition experiment of food samples (EW), a total R.S.D. of less than 6.75% and an average recovery between 95.45% and 102.62% were obtained.	[98], (2023)
EW (hydrogel) + (L-EVs)	Wound Dressing	Directional Lyophilization	The controlled and horizontal release of biochemical factors improved critical processes such as angiogenesis and collagen deposition.	[21], (2024)
Hydroxypropyl chitosan-EW hydrogel (HPCS-EWH)	Wound Dressing	Physical Crosslinking	It has been shown to reduce reactive oxygen species (ROS), exhibit antibacterial and anti-inflammatory activity, and support angiogenesis.	[57], (2023)
EW hydrogel scaffold (EWHS)	Scaffold	Three-Step Green Processing Technology	The tensile strength in wet state is as high as 5.0 MPa, the elongation at break is about 93%, and the swelling rate is about 85%. Biocompatible and biomaterial has potential applications in medical 3D tissue engineering materials, such as tendons, soft tissues, and in vivo implants.	[85], (2022)

## 6. Structure–Property–Function Relationships

The clinical and biomedical utility of EW-based biomaterials is determined not solely by their primary amino acid sequences or constituent bioactive components, but also by the complex interplay among processing-induced conformational changes, crosslinking mechanisms, and the resulting morphological and physicochemical characteristics. Accordingly, EW matrices display highly interdependent structure–property–function relationships; the ability to transform the same macromolecular precursor into a broad range of architectures, including isotropic hydrogels, cohesive films, electrospun nanofibrous meshes, colloidal nano- and microparticles, macroporous sponges, and cryogels, together with the distinct biological responses elicited by these platforms, supports this relationship [2,13] (Table 3).

At the molecular scale, network formation and consolidation are governed by a combination of hydrogen bonding, hydrophobic interactions, electrostatic associations, and sulfhydryl-disulfide exchange reactions. External stimuli, including thermal treatment, pH variation, and changes in ionic strength, promote the partial unfolding of native globular proteins, thereby exposing previously buried hydrophobic domains and reactive nucleophilic residues. Subsequent stabilization through chemical, enzymatic, or photochemical crosslinking generates permanent, load-bearing connections between polypeptide chains [16,22]. Although the reinforcement of intermolecular junctions enhances network elasticity, mechanical strength, and structural stability, it also reduces free volume and polymer segmental mobility. As a result, water retention, molecular diffusion, and hydrolytic degradation are concomitantly restricted.

Crosslinking density strongly affects the mechanical properties of EW-based materials. Reported stiffness values span a wide range, although they were obtained from materials in different physical states and with different test methods. Three-dimensionally printed, ionically crosslinked EW–alginate patches stored in PBS showed compressive elastic moduli of 20–27 kPa under unconfined compression [49], whereas poly-albumen films crosslinked with diethylenetriamine (DETA) and cured at 50 °C reached an elastic modulus of approximately 4 GPa under ambient conditions [42]. These values show that the stiffness of EW-based materials can range from soft, kPa-level hydrogels to stiff, GPa-level solid films. It should be noted that this difference arises not only from crosslinking chemistry but also from water content and test method. Accordingly, these values indicate the achievable stiffness range rather than the effect of crosslinking alone. The effect of crosslinking is more clearly seen within a single study using the same test method. For example, in dual crosslinked EW hydrogels, increasing the CaCl_2_ concentration from 0 to 2% (*w*/*v*) significantly increased hardness, as measured by texture analysis, but reduced porosity from 97.2% to 82.4% and decreased the equilibrium swelling ratio approximately sevenfold [38]. A comparable compromise exists between volumetric porosity and mechanical competence. Highly interconnected macroporous architectures facilitate cellular infiltration, vascular ingrowth, and the efficient transport of oxygen and metabolic substrates [85]. Nevertheless, excessive porosity can substantially compromise compressive strength and resistance to shear deformation. In EW-derived foams, sponges, and cryogels, macropores primarily serve as open pathways for parenchymal cell migration, whereas microporous interstitial walls contribute to capillary fluid retention and protein adsorption [56]. Thus, total porosity alone is an inadequate descriptor of scaffold performance. Pore size, orientation, interconnectivity, wall thickness, and wet-state mechanical properties must be evaluated collectively.

The dimensionality and morphology of an EW-based construct also exert a direct influence on its biological function. Highly hydrated hydrogels can reproduce the soft and viscoelastic characteristics of native tissues, making them suitable for cell encapsulation, moist wound management, and localized drug delivery, with post-printing cell viabilities exceeding 93% [48,49]. Electrospun nanofibers provide fibrillar topographical cues that resemble the structural features of the native collagenous extracellular matrix (ECM). Thin and cohesive films or membranes can function as microbial barriers, anti-adhesion interfaces, and biosensing substrates, whereas macroporous three-dimensional scaffolds, with macropores of 100–200 µm [56], are more appropriate for volumetric tissue regeneration. In colloidal systems, hydrodynamic diameter, surface zeta potential, and specific surface area represent key design parameters that regulate drug loading, systemic pharmacokinetics, and cellular internalization. As these values derive from different experimental conditions, they should be compared with caution.

Despite these advantages, the globular conformation of the principal EW proteins imposes important structural limitations. In contrast to fibrillar structural proteins such as collagen and silk fibroin, globular egg proteins do not form extended, entangled macromolecular chains in solution. This characteristic limits the viscoelasticity, chain entanglement, and stable jet formation required for the production of defect-free electrospun fibers [52]. The incorporation of synthetic or natural linear polymers, including poly(vinyl alcohol) (PVA), polycaprolactone (PCL), poly(ethylene oxide) (PEO), alginate, gelatin, and silk fibroin, can alleviate these limitations by improving chain entanglement, shear rheology, extrusion fidelity, and tensile performance.

Importantly, macromolecular hybridization does not improve all material properties in a uniform or unidirectional manner. For example, the incorporation of silk fibroin increased the compressive modulus and enzymatic stability of EW scaffolds [45]. In contrast, increasing the montmorillonite content in PVA/EW hydrogels enhanced network integrity and prolonged drug release, but simultaneously reduced the equilibrium water content and water vapor transmission rate of the dressings [41]. These observations challenge the simplistic assumption that secondary additives invariably improve biomaterial performance. Instead, their effects should be interpreted mechanistically by identifying the specific physicochemical parameters that are modified and determining how these changes influence the behavior of target cells.

Degradation kinetics constitute another critical component of the structure–property–function paradigm. In tissue engineering applications, scaffold resorption should be appropriately synchronized with the synthesis and maturation of newly deposited native ECM [12]. In drug delivery systems, matrix erosion directly influences release kinetics and, consequently, pharmacokinetic profiles [45]. Premature degradation may result in structural collapse and dose dumping, whereas excessively slow degradation can impede tissue remodeling and promote persistent foreign-body encapsulation. Accordingly, no single morphology can be regarded as universally optimal for EW-based biomaterials. Clinical performance instead depends on establishing an application-specific balance among mechanical resilience, architectural organization, fluid transport, degradation kinetics, and bioinstructive interfacial properties.

## 7. Current Advantages, Challenges, and Translational Perspectives

The translational potential of EW-based biomaterials is primarily attributed to their renewable natural abundance, broad accessibility, cost-effectiveness, and complex macromolecular composition. The simultaneous presence of ovalbumin, ovotransferrin, ovomucin, and lysozyme enables the integration of structural and biofunctional characteristics within a single naturally derived scaffold. For example, the intrinsic bactericidal activity of lysozyme against Gram-positive pathogens, together with the transition-metal-binding capacity of ovotransferrin, extends the potential applications of EW beyond those of an inert structural protein source [2].

From an economic and supply-chain perspective, EW represents an accessible, non-mammalian alternative to costly commercial basement membrane matrices, such as Matrigel, particularly for academic research and high-throughput applications. Nevertheless, crude EW should not be considered directly equivalent to Matrigel, which possesses a substantially more complex biochemical composition and well-defined basement membrane-associated signaling functions. Rather, EW is more appropriately regarded as a sustainable and chemically functionalizable protein-based platform for specific applications in cell culture, disease modeling, and tissue engineering [14].

EW-based systems also exhibit considerable versatility in terms of processing and fabrication. Depending on the intended application, these materials can be processed through thermal casting, pH-induced gelation, polyvalent cation-mediated complexation, cryotropic freezing, electrospinning, and free-radical- or photo-initiated crosslinking [2,46,60]. In addition, the optical transparency of certain EW-based formulations may facilitate real-time microscopic monitoring of cellular morphology, organization, and dynamic behavior in three-dimensional culture systems.

Despite these advantages, several important challenges must be addressed before the clinical translation of EW-based biomaterials can be achieved. One of the primary concerns is the biological variability of the source material. The genetic background of the chicken, dietary composition, animal age, egg storage duration, and processing conditions may all influence the protein composition and functional characteristics of EW. Consequently, substantial batch-to-batch variations may arise in gelation kinetics, viscosity, mechanical performance, and degradation behavior [14,60]. Because clinically relevant biomaterials require stringent and reproducible quality specifications, the use of standardized EW protein preparations or purified and compositionally defined protein fractions may become increasingly important.

Allergenicity and immunogenicity represent additional critical considerations. Several EW proteins, including ovalbumin and particularly ovomucoid, are recognized food allergens. However, the allergenic potential of these proteins following dietary exposure cannot be directly equated with the biological response elicited by a crosslinked or implanted EW-based biomaterial. Food-mediated allergen exposure and the interaction between an implanted proteinaceous material and the host immune system involve distinct exposure routes and biological mechanisms. Therefore, future studies should extend beyond conventional cytotoxicity assays and systematically investigate inflammatory cytokine secretion, macrophage polarization and activation, antigen-specific antibody production, and long-term local and systemic immune responses [2,14].

Sterilization and storage stability constitute further translational challenges. Because protein-based materials are susceptible to thermal, radiative, and chemical treatments, sterilization procedures may alter protein conformation, crosslinking density, network morphology, and mechanical properties. Accordingly, sterilization protocols should be evaluated not only in terms of their microbiological effectiveness but also with respect to their effects on the structural integrity, mechanical performance, degradation profile, and biological activity of the resulting biomaterial [9].

The mechanical limitations of EW-based materials, which are largely associated with the globular structure of their constituent proteins, are particularly relevant for load-bearing tissue applications and fiber-based constructs. To overcome these limitations, EW is frequently combined with polymers such as alginate, gelatin, poly(vinyl alcohol) (PVA), poly(ε-caprolactone) (PCL), and silk fibroin [3,48]. More advanced strategies have sought to simultaneously modulate mechanical performance and bioactivity through the incorporation of nanoscale reinforcing agents, including carbon quantum dots and silicate nanosheets, into the protein matrix [3,18]. Nevertheless, these approaches introduce additional safety considerations. In particular, the long-term biological effects of the incorporated nanomaterials and their potential release into the surrounding tissues during material degradation require systematic and long-term evaluation.

### Future Research Directions

Advanced biofabrication technologies are expected to play a central role in the development of future EW-based biomaterials. In particular, photocrosslinkable methacrylated EW derivatives enable rapid and spatially controlled crosslinking during extrusion-based bioprinting and light-assisted stereolithography. These properties facilitate the fabrication of anatomically complex scaffolds and perfusable vascular networks [16]. Nevertheless, future research should extend beyond the assessment of initial filament fidelity and systematically evaluate long-term phenotypic stability, vascular maturation, host–material integration, and functional tissue remodeling under dynamic physiological conditions.

4D bioprinting has considerable potential; however, EW-based materials have not yet found application in this field. Nevertheless, other protein-based biomaterials have been successfully produced using 4D printing. For instance, bovine serum albumin (BSA)-based hydrogels have exhibited not only reversible behaviors in response to temperature and pH changes but also permanent shape transformation via enzymatic degradation [99]. Similarly, in nanocomposites containing BSA and gold nanorods, near-infrared light-triggered shape recovery has been achieved based on the unfolding and refolding of protein chains [100]. The ability to convert EW into photocrosslinkable derivatives [16,44] and to fabricate adaptive structures from EW hydrogels [51] indicates that these approaches could also be adapted to EW. For such an adaptation, the reproducibility, kinetics, and biological significance of the resulting dynamic change must be experimentally verified.

Another research direction concerns the development of more advanced stimuli-responsive drug-delivery systems. Engineering EW-based matrices capable of selectively modulating their permeability or degradation kinetics in response to local microenvironmental cues, such as elevated matrix metalloproteinase activity, localized acidosis within tumor niches, or oxidative stress in chronic wounds, could enable precise and on-demand therapeutic release [64]. However, the translational potential of these systems will depend on demonstrating that their stimulus-responsive behavior remains robust in complex physiological fluids and tissue environments rather than being limited to simplified in vitro buffer systems.

A further strategic priority is the transition from the use of whole raw EW toward purified and fraction-specific formulations. Although existing studies have largely focused on whole EW or purified ovalbumin [2,11], the selective isolation and rational recombination of individual protein fractions could enable the design of function-specific biomaterials with improved reproducibility and reduced batch-to-batch variability. For example, the iron-chelating capacity of ovotransferrin, the interfacial stabilization and foaming properties of ovomucin, and the bacteriolytic activity of lysozyme could be systematically integrated to generate biomaterial platforms tailored to specific biomedical applications [10].

Finally, although raw EW is renewable, widely available, and economically attractive, its overall environmental and economic sustainability cannot be inferred solely from its low-cost and bio-based origin. The complete industrial life cycle of EW-derived biomaterials, including energy-intensive protein isolation, lyophilization, ultraviolet curing, and specialized sterilization procedures, should therefore be assessed through rigorous life-cycle assessments (LCAs). Such analyses will be essential for determining whether these materials can maintain their apparent ecological and economic advantages when produced at manufacturing scale [14].

## 8. Conclusions and Perspectives

EW is increasingly recognized as a versatile and renewable protein-based biomaterial platform with potential applications in tissue engineering, regenerative medicine, wound healing, drug delivery, and bioelectronics. Its major proteins, including ovalbumin, ovotransferrin, ovomucoid, ovomucin, and lysozyme, provide complementary structural and biological functions that can be exploited for biomaterial design [2,4,13]. Importantly, EW functionality is determined not only by protein composition but also by protein conformation, intermolecular interactions, and structural organization during processing.

The reviewed studies demonstrate that EW can be processed into diverse architectures, including hydrogels, cryogels, fibrous matrices, films and membranes, porous scaffolds, and particulate systems. These architectures provide distinct properties suited to different biomedical functions, ranging from three-dimensional cell support and tissue regeneration to wound coverage and controlled therapeutic delivery. This versatility highlights the importance of selecting material architecture according to the intended biological function rather than considering EW composition alone.

A central conclusion of this review is that EW-based biomaterial performance is governed by a multiscale processing, structure, property, and function relationship. Processing conditions regulate protein denaturation, aggregation, crosslinking, and functional-group exposure, which in turn determine hydration, stiffness, porosity, permeability, degradation, and bioactive ligand presentation. Consequently, similar protein compositions may elicit substantially different biological responses when organized into different material architectures. Establishing quantitative relationships among these parameters should therefore be a priority for future studies.

Several challenges must nevertheless be addressed before clinical translation. Natural-source variability may influence material composition, gelation, degradation, immunological behavior, and biological performance, emphasizing the need for standardized formulations and rigorous batch characterization. Sterilization methods must also be optimized to preserve protein conformation and bioactivity. Furthermore, potential immunogenicity, limited mechanical robustness, and long-term instability require systematic investigation. Crosslinking and hybrid-network strategies may address some of these limitations, provided that cytocompatibility, porosity, biodegradability, and intrinsic bioactivity are maintained.

Overall, future research should move beyond proof-of-concept demonstrations toward predictive, standardized, and application-specific biomaterial design. Integrating protein composition, processing conditions, molecular organization, material architecture, and biological outcomes will be essential for establishing EW as a reliable, safe, and clinically translatable biomaterial platform.

## Figures and Tables

**Table 3:** Relationship between morphological architectures, fabrication strategies, structural properties, and biomedical applications of EW-based biomaterials.

Morphology	Fabrication Strategy	Property Features	Biomedical Applications
Hydrogels	Physical gelation; chemical gelation; enzymatic gelation; photocrosslinking; hybridization	Hydrated three-dimensional networks, tunable swelling, viscoelastic properties, and degradation behavior	Wound healing, 3D Cell Culture, Tissue Engineering, Drug Delivery, 3D Bioprinting
Films/Membranes	Physical gelation; chemical gelation; enzymatic gelation; photocrosslinking; hybridization	Continuous structures, tensile properties, flexibility, and barrier behavior	Wound Dressings, Protective Membranes, Biointerfaces
Micro-/Nanoparticles	Physical gelation; chemical gelation; enzymatic gelation; hybridization	High surface-to-volume ratio, tunable particle size, and diffusion-dependent release	Drug and Bioactive Molecule Delivery, Biosensing
Micro-/Nanofibers	Chemical gelation; hybridization	Fibrous architectures, high specific surface area, and tunable mechanical properties	Wound healing, Tissue Engineering Scaffolds, Bioactive Membranes
Porous Sponges/Cryogels	Chemical gelation	Interconnected macropores, high water uptake, compressibility, and permeability	3D Cell Culture, Tissue Engineering, Wound Healing
3D-Printed Constructs	Physical gelation; enzymatic gelation; photocrosslinking	Controlled geometries, interconnected porous architectures, and tunable shape fidelity	3D Bioprinting, Tissue Engineering, Wound Healing

## Data Availability

No new data were created or analyzed in this study. Data sharing is not applicable to this article.
